# Golgi fucosyltransferase 1 reveals its important role in α-1,4-fucose modification of N-glycan in CRISPR/Cas9 diatom *Phaeodactylum tricornutum*

**DOI:** 10.1186/s12934-022-02000-2

**Published:** 2023-01-07

**Authors:** Xihui Xie, Jianchao Yang, Hong Du, Jichen Chen, Edmond Sanganyado, Yangmin Gong, Hua Du, Weizhou Chen, Zhengyi Liu, Xiaojuan Liu

**Affiliations:** 1 Guangdong Provincial Key Laboratory of Marine Biotechnology, Guangdong Provincial Key Laboratory of Marine Disaster Prediction and Prevention, Institute of Marine Sciences, STU-UNIVPM Joint Algal Research Center, College of Sciences, Shantou University, Shantou, 515063 Guangdong China; 2 Yantai Academy of Agricultural Sciences, Yantai, 265500 Shandong China; 3 Yantai Institute of Coastal Zone Research, Chinese Academy of Sciences, Yantai, 264003 Shandong China

**Keywords:** *Phaeodactylum tricornutum*, Glycoprotein, Fucosyltransferase, N-glycan, Microbial cell factory

## Abstract

*Phaeodactylum tricornutum* (Pt) is a critical microbial cell factory to produce a wide spectrum of marketable products including recombinant biopharmaceutical N-glycoproteins. N-glycosylation modification of proteins is important for their activity, stability, and half-life, especially some special modifications, such as fucose-modification by fucosyltransferase (FucT). Three PtFucTs were annotated in the genome of *P. tricornutum*, PtFucT1 was located on the medial/trans-Golgi apparatus and PtFucT2-3 in the plastid stroma. Algal growth, biomass and photosynthesis efficiency were significantly inhibited in a knockout mutant of PtFucT1 (PtFucT1-KO). PtFucT1 played a role in non-core fucose modification of N-glycans. The knockout of PtFucT1 might affect the activity of PtGnTI in the complex and change the complex N-glycan to mannose type N-glycan. The study provided critical information for understanding the mechanism of protein N-glycosylation modification and using microalgae as an alternative ecofriendly cell factory to produce biopharmaceuticals.

**Supplementary Information:**

The online version contains supplementary material available at 10.1186/s12934-022-02000-2.

## Introduction

Protein N-glycosylation (Asn-linked glycosylation) is an important posttranslational modifications that often alter the physicochemical properties of the proteins (e.g., protein folding, activity, secretion and intracellular trafficking, and cell–cell communication) in eukaryotic cells [1]. It is initiated by the biosynthesis of Man_5_GlcNAc_2_ glycan linked to dolichol pyrophosphate (PP-dolichol) on the cytosolic side of the ER membrane. The glycan subsequently flips into the ER lumen side and continues to yield the N-glycan precursor, Glc_3_Man_9_GlcNAc_2_-PP-dolichol. The precursor is then transferred to the consensus Asn-Xaa-Ser/Thr motif (X not proline) of nascent proteins by the oligosaccharyltransferase (OST) complex [2]. After removing two glucose residues the glycoprotein is recognized by ER-resident chaperones, such as calnexin and calreticulin, to ensure the quality control of the synthesized protein. Following quality control, correctly folding glycoproteins are exported to the Golgi for further modifications. Incorrectly folding glycoproteins are re-glucosylated and quality controlled by ER-resident chaperones or degraded by ER-associated degradation (ERAD) mechanism. Such ER events are highly conserved in most eukaryotes investigated to date [3]. In contrast, N-glycan processing in the Golgi apparatus is organism-specific and produces a wide array of complex N-glycans under different glycotransferases and glycosidases, such as fucosyltransferase (FucT) and xylosyltransferase (XylT) [4, 5]. These organism-specific modifications yield different types N-glycan structures, including high mannose, complex, hybrid, and paucimannose types [1].

In recent years, several studies investigated the types, subcellular localization, and functions of fucosyltransferase (FucT) in different eukaryotes, including animals and plants [6]. Fucosyltransferase is responsible for the transfer of fucose residue from GDP-fucose to the N-glycan of neosynthesized glycoproteins during the N-glycosylation. The FucT on the fucose modification on a core N-acetylglucosamine (GlcNAc) is called core FucT while that on a non-core GlcNAc is a non-core FucT. About 13 FucTs (FUT1-13) which functioned as α-1,2, α-1,3, α-1,4 and α-1,6-FucTs were identified in animals [7]. The core FUT8, which facilitates the modification on the α-1,6 site of core GlcNAc by fucose residue, plays a critical role in immune system function and growth and development [8]. While 13 FucTs (AtFucT1-13) were also identified in *Arabidopsis thaliana*, AtFucT1-10, which belongs to the α-1,2-FucTs, was the FucT that participated in the fucose modification of cell wall glycol-complexes [9, 10]. AtFucT11-12, core α-1,3-FucTs located in the medial or trans-Golgi apparatus, play an important role in salt stress resistance [11]. In contrast, AtFucT13, a α-1,4-FucT located in the trans-Golgi apparatus, participate in the synthesis of Lewis a-type glycans [12]. In addition to *A. thaliana*, FucTs were also identified from other plants, such rice (*Oryza sativa*) and Japanese trefoil (*Lotus japonicus*) where they played a role in the transport of auxin, the growth and development of plants, and immune defense [13, 14]. A previous study found that FucT in *Chlamydomonas reinhardtii* transferred fucose residues onto N-glycans in a core xylose dependent manner [15].

Interest on the expression of biopharmaceuticals in microalgae and plants has grown significantly in the past few decades due to an increasing need for environmentally sustainable production processes that are also economically feasible [16]. Previous studies describe the expression of recombinant N-glycoprotein biopharmaceuticals in the chloroplast and/or nucleus of seven microalgae species [17]. Since the expression of functional monoclonal antibodies in transgenic tobacco in 1989, more biopharmaceutical relevant proteins were produced in different plant tissues worldwide [18]. Plant-derived biopharmaceutical glycoproteins such as Elelyso produced in carrot cells and ZMapp antibody produced in *Nicotiana benthamiana* have been clinically trialed for treating Gaucher’s disease and Ebola virus, respectively [17]. The wide differences between plant and mammalian N-glycan structures limit the development of plant-derived biopharmaceuticals for use in human therapy [19]. In addition, biopharmaceutical glycoproteins produced by plants are modified by specific α-1,3-fucose and/or β-1,2-xylose residues, which are potentially allergens in humans [19]. Recent years, microalgae, such as diatom *Phaeodactylum tricornutum*, were used for a new alternative biopharmaceutical producer [17]. Anti-hepatitis B IgG and anti-MARV NP IgG were expressed in *P. tricornutum*, and it was shown that N-glycosylation modification was important for the function of these biopharmaceuticals. Hence, understanding the function of the key gene FucT is imperative for the humanization of the microalgal glycosylation pathway and the production of functional biopharmaceutical N-glycoproteins in microalgae.

Previous studies showed that among the three FucT coding sequences identified in *P. tricornutum* (ID: 54599, 46109 and 46110), the overexpression of FucT (ID:54599) increased the core α-1,3-fucose modified N-glycoproteins [20]. However, the consequences in physiological, N-glycoproteomic and N-glycomic levels in *P. tricornutum* remain unknown. Based on the bioinformatic analyses, we hypothesized that three potential PtFucTs in *P. tricornutum* locate to Golgi apparatus and participate in the fucose modification of N-glycans. In this study, comprehensive approaches including bioinformatic, physiological, N-glycoproteomic, and N-glycomic analyses were used to investigate the functions of putative fucosyltransferases (PtFucTs) in the model diatom *P. tricornutum*. The results revealed that medial/trans-Golgi located PtFucT1 participated in the non-core α-1,4-fucose modification of N-glycans, interacted with PtGnTI and affected the synthesis of complex type N-glycans. PtFucT2 and PtFucT3 proteins were targeted to the plastid of *P. tricornutum*, however, their function was still unknown. In this study, the analysis of N-glycan structures and its biosynthesis pathway will be valuable for studying glycoscience and pharmaceuticals.

## Experimental procedures

### Cultivation of *P. tricornutum* and biolistic transformation

*Phaeodactylum tricornutum* Pt1 wild type, PtFucTs overexpression and PtFucT1 knockout mutants were cultured in f/2 medium at 22 ℃ under 24 h light condition (50 μmol photons m^−2^ s^−1^ white light) [21]. 1.3% of Agar–Agar Kobe I was used as a solid medium for *P. tricornutum*. The biolistic transformation was carried out as described previously [22]. The transfected clones were selected under the 75 μg/ml zeocin™ (Tiangen Biotech Co., China) for about three weeks. NH_4_Cl was used as nitrogen source under the non-induced conditions, while NaNO_3_ was used as the sole nitrogen source to induce the expression of PtFucTs-eGFP and cas9 proteins [23]. The expression of PtFucTs-eGFP was induced for 2 days and the expression of cas9 was induced for 7 days on the solid medium.

### Bioinformatic analysis of the three PtFucTs

The known PtFucT proteins were used to screen *P. tricornutum* genome in an online database [3]. Annotations of the predicted gene models were compared with the expressed sequence tags (EST) data retrieved from the NCBI database. The core FucT was identified from the genome database (https://mycocosm.jgi.doe.gov/pages/search-for-genes.jsf?organism=Phatr2 and http://protists.ensembl.org/Phaeodactylum_tricornutum/Info/Index?db=core). SignalP 5.0 (https://services.healthtech.dtu.dk/service.php?SignalP-5.0) was used to predict potential signal peptides, while TargetP (https://services.healthtech.dtu.dk/service.php?TargetP) predicted the targeting peptides. Transmembrane domain was predicted by different servers, including TMHMM Server v. 2.0 (http://www.cbs.dtu.dk/services/TMHMM-2.0/), DeepTMHMM (https://services.healthtech.dtu.dk/service.php?DeepTMHMM), TOPCONS (http://topcons.cbr.su.se/) and SOSUI (http://harrier.nagahama-i-bio.ac.jp/sosui/sosui_submit.html). The functional domain was primarily clarified by two servers Pfam (http://pfam.xfam.org/) and SMART (http://smart.embl.de/). Furthermore, the subcellular localization of proteins was predicted by Cell-Ploc 2.0 (http://www.csbio.sjtu.edu.cn/bioinf/Cell-PLoc-2/), HECTAR v1.3 (http://www.sb-roscoff.fr/hectar/) and WoLF PSORT (https://wolfpsort.hgc.jp/). Further information on the N-glycoproteins was retrieved from the SWISS-MODEL (https://swissmodel.expasy.org/). The length of amino acids were analyzed online (https://www.uniprot.org/, http://protists.ensembl.org/Phaeodactylum_tricornutum/Info/Index?db=core and https://mycocosm.jgi.doe.gov/pages/search-for-genes.jsf?organism=Phatr2).

Full-length of FucT proteins were aligned by ClustalW software with default parameters [24]. The unrooted phylogenetic tree was constructed by MEGA 6.0 software with 1,000 bootstrap replicates via the Neighbor-Joining tree method with default parameters [25]. The tree was subsequently improved by Evolview (https://evolgenius.info//evolview-v2/#login). The conserved motifs of FucTs from land plants and *P. tricornutum* were also aligned by ClustalW.

### Generation of vectors for eGFP fusion genes and in vivo localization

RNA isolation and cDNA synthesis were carried out according to standard procedures [26]. PtFucTs sequences were fused to the upstream of eGFP and cloned into pPha-NR vector (GenBank: JN180663) via EasyGeno kit (Tiangen, Biotech Co., China). During the fusion, the stop codon of PtFucTs was deleted. The primers were designed via the EasyGeno Primer website (http://123.56.75.195/#enidx). pPha-NR-PtFucTs-eGFP plasmids were transfected into *P. tricornutum* cells on the 5th day of culture. Afterwards, the clones were analyzed with confocal laser scanning microscope (LSM 800 Meta, Carl Zeiss, Germany). An argon laser at 488 nm was used to activate the fluorescence of eGFP and plastid autofluorescence. While the fluorescence was detected at bandwidths of 500–520 nm (green fluorescence of eGFP), 580–600 nm (magenta fluorescence of mRFP) and 625–720 nm (red fluorescence of plastid), respectively [21]. All primer sequences used in this paper were shown in Additional file 2: Table S1.

### Generation of vectors for Crispr/Cas9 and verification of PtFucT1 mutants

Online software Benchling (https://benchling.com/) was used to design gRNA candidates with high on-target and off-target scores. Selected gRNAs were cloned into the ptCC9 (MH143578) using annealed synthetic oligomers and BsaI digested ptCC9 as the introduction of the previous paper [23]. The ptCC9-gRNAs were transfected into the wild type *P. tricornutum* cells. The clones were then transferred to the inducing medium for seven days. Afterwards, colony PCR and sequencing of the amplicons were performed to identify the potential mutants. If the sequences in the targeting region were ambiguous, the clones were cultured in the liquid f/2 medium and spread on agar plates to obtain single clones. The raw sequencing data of these isolated mutants were analyzed by an online-based software ICE v2 CRISPR analysis tool [27]. At the same time, T7 endonuclease I assay (New England Biolabs) was used to determine the efficiency of Cas9 mutagenesis [28]. Additionally, the expression of the potential PtFucT mutants were analyzed by PCR and quantitative real-time PCR (qRT-PCR) kit (Tiangen, Biotech Co., China). Each reaction mixture (20 μL) included ddH_2_O 7.4 μL, forward primer 0.8 μL, reverse primer 0.8 μL, cDNA 1 μL, and SuperMix 10 μL. The thermocycle program for each qRT–PCR reaction was as follows: 95 °C for 10 min, followed by 95 °C for 10 s, 60 °C for 20 s, 72 °C for 25 s, 40 cycles. Three biological and technical replicates were performed in this study, respectively. Six-serial dilutions of first-chain cDNA template were used to determine the standard curve of qRT-PCR. The comparative threshold (2^−△△Ct^) method was used to measure the relative expression levels [29]. 30S ribosomal protein subunit (RPS) gene was used as a housekeeping gene [30].

### Analysis of physiological characteristics

Cell density of *P. tricornutum* (1 mL) was detected using an electronic particle counter (Orifice, 50 µm; Multisizer II; Beckham Coulter, Fullerton, CA, United States) [31]. 30 mL algae were collected by Whatman GF/C (1.2 μm). The biomass was calculated by the formula: Biomass (g/ L) = (G2–G1) × 1 L/0.3 L, where G1 was the weight of dry Whatman (g) and G2 the weight of dry Whatman containing algae (g). The maximum quantum yields (Fv/Fm) were measured with a portable pulse amplitude modulated fluorometer Water-PAM (Walz, Effeltrich, Germany). The algal cells (2 mL) were cultured in the dark for 15 min to achieve completely oxidative PSII reaction centers before the measurement [29]. Chlorophyll a content was analyzed by 80% methanol and quantified by UV–VIS spectrophotometer at 652, 665 and 750 nm [32]. The soluble polysaccharides were analyzed by Micro Plant Soluble Sugar Content Assay Kit (NanJing JianCheng Bioengineering Institute). The measurement was carried out using UV–VIS spectrophotometry at 620 nm [33]. The analysis of soluble protein was performed by BCA protein assay kit (Beyotime Biotechnology, China) using UV–VIS spectrophotometry at 595 nm [34].

Statistical analyses were performed with SPSS 25.0 for windows. The difference was analyzed by one-way ANOVA followed by Duncan’s multiple range tests. A value of *P* = 0.05 was considered as statistically significant, while a value of *P* = 0.01 was considered as statistically extremely significant. All data were calculated with three biological replicates and reported as the means ± SD.

### N-glycoproteome of PtFucT1 mutants

2 g (fresh weight) *P. tricornutum* cells were ground prior to protein extraction, acetone precipitation, and protein denaturation. Protein extraction was carried out based on previous procedures described in detail by Hao et al. [35]. The protein concentration was determined by BCA Protein Assay Kit (Sigma) according to the manufacturer’s guidelines [35]. Subsequently, 1 mg protein was reduced with 10 mM Tris (2-carboxyethyl) phosphine at 55 °C for 1 h, alkylated with 20 mM iodoacetamide in the dark at room temperature for 30 min, and digested with trypsin 1:50 w/w at 37 °C for 16 h. The digestants were then desalted by on C18 SPE tips and eluted sequentially with acetonitrile with trifluoroacetic acid. The eluants were finally mixed and dried on a SpeedVac. Intact N-glycopeptides were enriched using ZIC-HILIC SPE tips [36].

PNGase enzymes were used for de-glycosylation of N-glycopeptides [38]. The peptides were subsequently divided into four fractionations and dried with vacuum concentration meter. LC–MS/MS data acquisition was carried out on an Orbitrap Exploris 480 mass spectrometer coupled with an Easy-nLC 1200 system. Peptides were loaded through auto-sampler and separated in a C18 analytical column (75 μm × 25 cm, C18, 1.9 μm, 100 Å). Mobile phase A (0.1% formic acid) and mobile phase B (80% acetonitrile, 0.1% formic acid) were used to establish the separation gradient. A constant flow rate was set at 300 nL/min. For Data-Dependent Acquisition (DDA) mode analysis, each scan cycle is consisted of one full-scan mass spectrum (R = 60 K, AGC = 300%, max IT = 20 ms, scan range = 350–1500 m/z) followed by 20 MS/MS events (R = 15 K, AGC = 100%, max IT = auto, cycle time = 2 s). HCD collision energy was set to 30. Isolation window for precursor selection was set to 1.6 Da. Former target ion exclusion was set for 35 s.

The Isotopic labeling method was used for the high-throughput sequencing. Tandem mass spectra were searched against the protein database of *P. tricornutum* (Uniprot: https://www.uniprot.org/, Release 2022_05 and http://protists.ensembl.org/Phaeodactylum_tricornutum/Info/Index) (M15095v2) [39]. All the searched protein sequences were used as parameter for the next analysis. 20 was set as the minimum number of occurrences. Emulate original motif-x was ticked, and other parameters with default. In addition, InterProScan (v.5.14–53.0) was used for the annotations of GO and Domain [40]. KAAS (v.2.0) and KEGG Mapper (V2.5) were used for the annotation of Kyoto Encyclopedia of Genes and Genomes (KEGG) [41]. The analysis of enrichment was performed via the Perl module (v.1.31) [42].

### N-glycan structure analysis of PtFucT-KO mutant

The protein extraction, trypsin digestion and N-glycopeptides enrichment were performed as the description in 2.6.1. After the enrichment of the digested N-glycopeptides by ZIC-HILIC SPE tips, the intact N-glycopeptides from wild type and PtFucT-KO mutant were reductively di-ethylated with acetaldehyde and acetaldehyde-^13^C_2_ [43, 44]. 100 μL 2,2,2-trifluoroethanol was added to all the intact N-glycopeptides and then 0.25 μL 20% CH_3_CHO (or ^13^CH_3_^13^CHO) per 1 μg glycopeptides and 600 mM NaBH_3_CN were added followed by immediate vortex for 1 min. The mixture was incubated at room temperature for 1 h and quenched with 4% NH_4_OH. The isotopic labelled intact N-glycopeptides were desalted by a C18 SPE column and dried, finally dissolved in ultrapure water for LC–MS analysis. The whole procedures was carried out as the description in the previous paper [45].

The C18-RPLC-MS/MS (HCD) analysis was carried out by analysis column: 360 μod × 75 μid, 75 cm long with C18 particles (Phenomenex, Jupiter, 300 Å, 5 μm) and trap column: 360 μod × 200 μid, 5 cm long with C18 particles (Phenomenex, Jupiter, 300 Å, 5 μm). The flow rate of sample loading was controlled at 5 μL/min, the analysis at 300 nL/min. Temperature of the ion transfer tube was 300 ℃, and spray voltage was 1.9 kV. The automatic gain control (AGC) target value was 3 × 10^6^, while the maximum injection time was placed at 20 ms. The MS spectra of 700−2000 m/z with mass resolution 60 k (m/z 200) were obtained for the next analysis. The mass resolution was set at 30 k for MS/MS spectra. Fragmentation was obtained in a DDA Top20 using HCD with stepped NCE (20%, 30%, 31%). The AGC target value and maximum injection time were placed at 5 × 10^5^ and 250 ms. Isolation window and dynamic exclusion were set at 3.0 m/z and 20.0 s. The resulting MS/MS data were processed using Maxquant (1.6.15.0) search engine and GPSeeker [46].

### Yeast two-hybrid assay

The full-length CDS of PtFucT1 was amplified and inserted into the vector pBT3-N to generate a bait plasmid. The CDS of PtGnTI gene was then fused into the vector pPR3-N to generate a prey plasmid. The bait and prey plasmids were transformed into NMY51 strain. The protein interaction was assessed by the growth of strain on the DDO[SD/-Leu/-Trp], TDO[SD/-Leu/-Trp/-His] and QDO[SD/-Leu/-Trp/-His/-Ade] mediums. The strain can growth on DDO medium, indicating that two plasmids were successfully transformed into the strain and non-toxic to strain. The strain can growth on TDO, but not on QDO, suggesting that the two proteins can interact. The more details were introduced in a previous paper [47].

## Results

### Bioinformatic analysis and subcellular localization of three PtFucTs

**Fig. 1:**
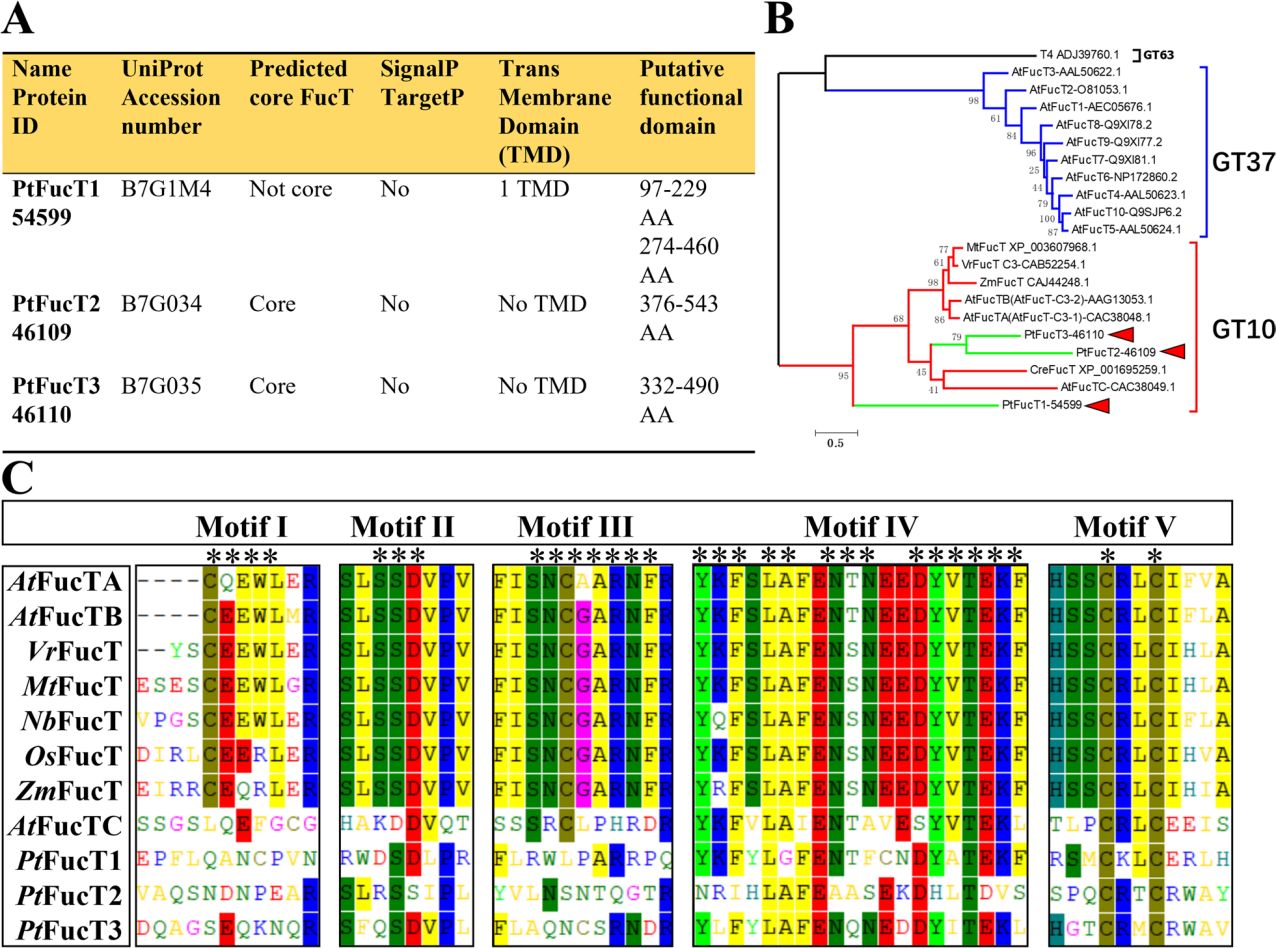
Bioinformatic analysis of three PtFucTs. **A** the predicted core FucT, signal peptide, targeting peptide, transmembrane domain and putative functional domain of the three PtFucTs; AA, amino acids; **B** the phylogenetic relationships of FucTs. GT, glycosyltransferase. Neighbor-joining (NJ) tree with 1000 bootstrap replicates was constructed based on the full-length sequences of FucTs using MEGA 6.0 software and online EvolView. The three red triangles are the three PtFucTs from *P. tricornutum*. The numbers in the phylogenetic tree are used to retrieve the protein sequences from the NCBI database (https://www.ncbi.nlm.nih.gov/). **C** the conserved motifs of FucTs from land plants and *P. tricornutum* by ClustalW. Motif I to Motif V are five conserved motifs of core FucTs in land plants

Three PtFucT gene models (ID:54599, PtFucT1; ID:46109, PtFucT2; and ID:46110, PtFucT3) were provided by the genome database of *P. tricornutum* (https://mycocosm.jgi.doe.gov/pages/search-for-genes.jsf?organism=Phatr2) and supported by EST data ((http://blast.ncbi.nlm.nih.gov/Blast.cgi?PROGRAM=tblastn&PAGE_TYPE=BlastSearch&LINK_LOC=blasthome) (Fig. 1A). Corresponding 481, 770 and 718 amino acids were subsequently encoded, the accession numbers in UniProt were B7G1M4, B7G034 and B7G035. Based on the protein sequences from the UniProt database, PtFucT1 was annotated to a non-core fucosyltransferase with one transmembrane domain, while PtFucT2 and PtFucT3 were annotated to core fucosyltransferases without a transmembrane domain. The protein sequences of PtFucTs were used to predict the functional domains. PtFucT1 contained two putative fucosyltransferase domains (PF00852) while PtFucT2 and PtFucT3 possessed a putative fucosyltransferase domain.

The phylogenetic analysis of FucT full-length sequences from different organisms (*Arabidopsis thaliana*, *Chlamydomonas reinhardtii*, *Vigna radiata* and *Medicago truncatula*) was shown in Fig. 1B. AtFucT1-10 from *A. thaliana* belonged to the GT37 family of non-core FucTs, while AtFucTA-C and other FucTs from *A. thaliana*, *C. reinhardtii*, *V. radiata* and *M. truncatula* belonged to GT10 family of core FucTs. Glucosyltransferase (T4, ADJ39760.1) from bacteriophage, belonging to GT63 family, was used as a control (out group). Fucosyltransferases catalyze the transfer of GDP-Fucose to different substrate acceptors. They are classified into 9 glycosyltransferase (GT) families in the carbohydrate active enzymes database (CAZy) [9]. Our results showed that the phylogenetic tree had three independent clades, belonging to GT63, GT37 and GT10. GT63 contained a bacteriophage T4 while GT37 contained 10 FucTs from *A. thaliana*, (AtFucT1-10). However, the three PtFucTs from *P. tricornutum* were in the GT10 clade together with FucTs from land plants and *C. reinhardtii*. Multiple sequence alignment showed that FucTs from land plants had five conserved motifs (Motif I to Motif V), while the three PtFucTs from *P. tricornutum* only contained two conserved motifs (Motif IV and V) (Fig. 1C). Motif IV and V were located inside the second putative functional domains of PtFucT1 and the domains of PtFucT2 and PtFucT3.

**Fig. 2:**
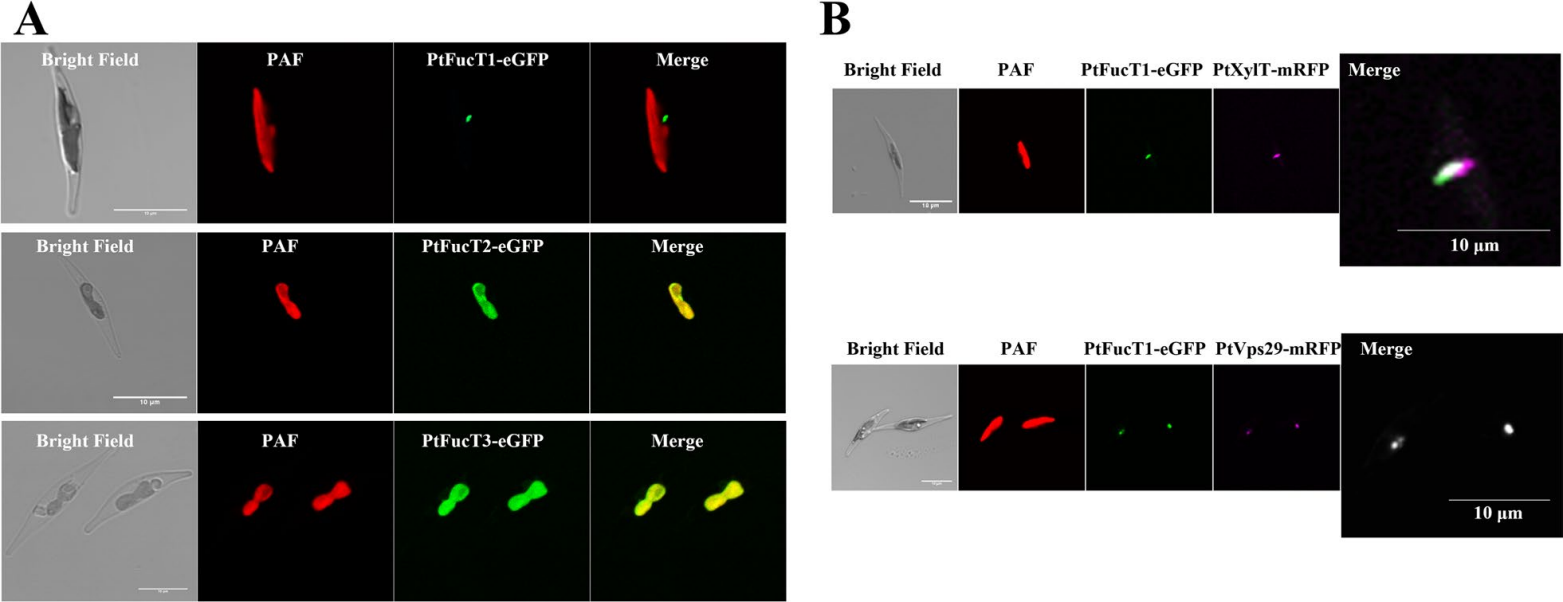
In vivo subcellular localization of the three PtFucTs. **A** the fluorescent pictures of PtFucT1-3-eGFP in *P. tricornutum*; **B** the co-localized fluorescent pictures of PtFucT1-eGFP and PtXylT-mRFP, PtFucT1-eGFP and PtVps29-mRFP, respectively. The co-localization data were referenced from the Ph.D thesis and published paper of the first author Xiaojuan Liu [21]; Bright Field, the cells under the transmitted light; PAF (red), plastid autofluorescence; eGFP (green), enhanced green fluorescence protein; mRFP (magenta), monomeric red fluorescent protein; Merge, the merge of PAF and eGFP

The N-glycosylation pathway of protein starts in the ER and continues in the Golgi apparatus until maturation. The predictions of subcellular localization indicated that PtFucT1 might be in the ER (WoLF PSORT) or plastid (Cell-Ploc 2.0), PtFucT2 in the plastid or vacuole (WoLF PSORT), and PtFucT3 in plastid (WoLF PSORT, HECTAR v1.3 and Cell-Ploc 2.0). Subsequently, the predictions were further clarified by experiments. Fusing the three PtFucTs with the eGFP at their C-terminus, revealed that the fluorescence of PtFucT1-eGFP accumulated in the central part of the cells next to the plastid (Fig. 2A). The diatom *P. tricornutum* cell contains a textbook-like Golgi apparatus consisting of cis-, medial- and trans-Golgi cisternae [21]. The pattern of PtFucT1-eGFP fluorescence was similar to the Golgi cisternae (Fig. 2A). However, the eGFP fluorescence of PtFucT2 and PtFucT3 overlapped with the autofluorescence of plastid and had a plastid pattern (Fig. 2A). Hence, the eGFP fluorescence detections of the three PtFucTs were not completely consistent with in silico predictions of subcellular localization, it was suggested that the predictions need experiments to confirm.

Additionally, the localization of PtFucT1-eGFP was further confirmed by the co-localization, as shown in Fig. 2B. It was shown that the green fluorescence of PtFucT1-eGFP was partially co-localized with the magenta fluorescence of medial Golgi marker PtXylT-mRFP, and completely co-localized with the magenta fluorescence of trans-Golgi marker PtVps29-mRFP.

### Verification of PtFucT1 knockout mutant and its physiological characteristics

**Fig. 3:**
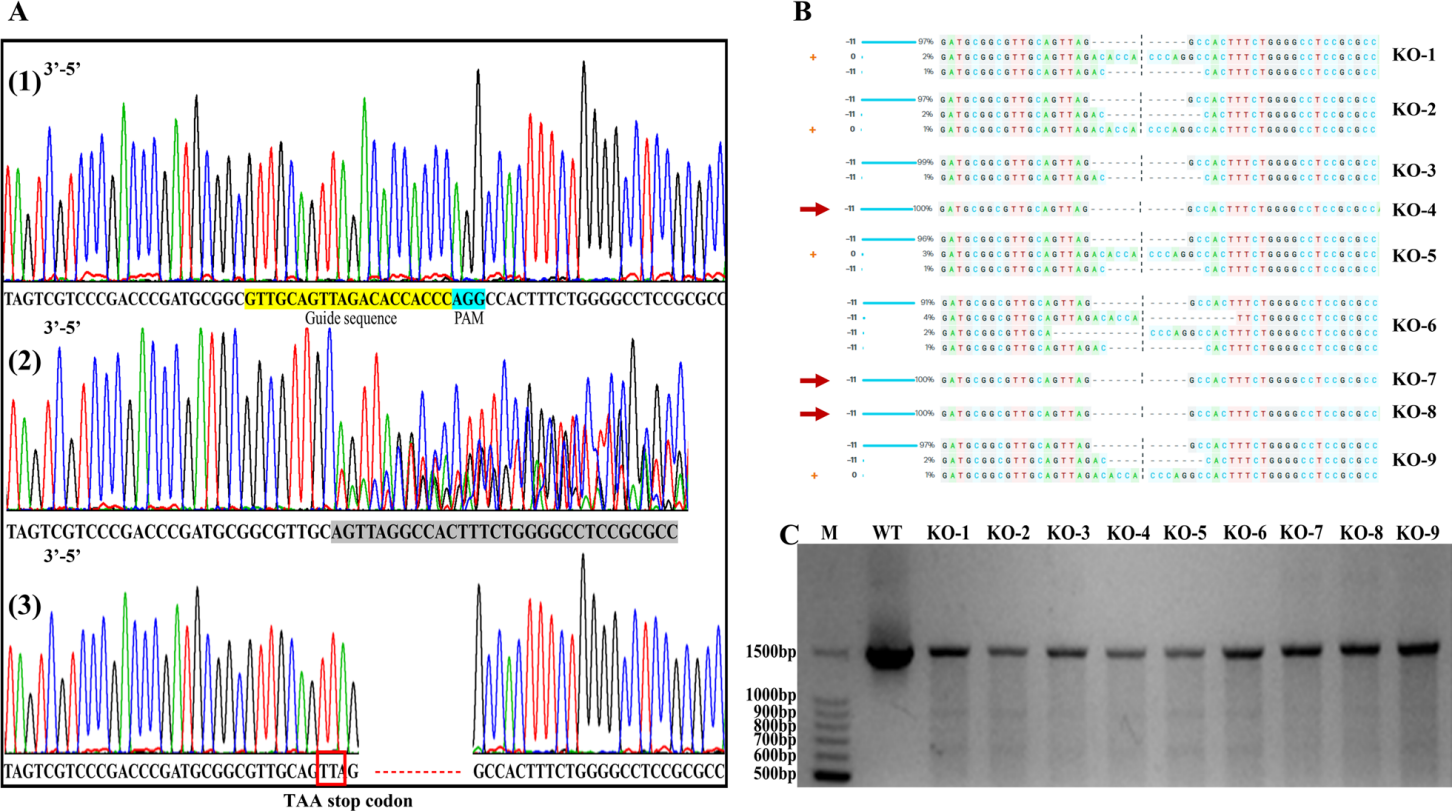
Knockout mutants of PtFucT1. **A** chromatogram of the wild type PtFucT1 gene (1), clone expression ptCC9 with the gRNA gene (PtFucT1-836) (2) and the isolated PtFucT1 knockout mutant with 11 nucleotides deletion (3); **B** the sequences of 9 potential knockout mutants were certificated by online ICE; **C** the 9 potential knockout mutants were verified by T7 endonuclease

A sole Golgi-like fluorescence pattern was observed from PtFucT1. We constructed PtFucT1 knockout mutant using Crispr/Cas9 genome editing technology to determine whether inactivation of PtFucT1 influenced the N-glycosylation of proteins in *P. tricornutum*. Compared to the chromatogram of wild type (Fig. 3A-1), PtFucT1 mutant with equivocal sequences in the targeting region was observed, as shown in Fig. 3A-2. This clone was subsequently cultured and used to isolate single clones in solid f/2 medium. Twenty-two single clones were picked out and sequenced in the targeting region, of which nine clones had clear chromatograms showing 11 missing nucleotides, and this directly stopped the open reading frame by the stop codon TTA (3′–5′, marked by red box) (Fig. 3A-3). Therefore, the functional Motifs IV and V were completely knocked out. Two clones had clear chromatograms with three nucleotides missing (data not shown). The missing three nucleotides (CCT) led to the knockout of one amino acid (arginine) on the open reading frame of PtFucT1. Subsequently, the 9 clones were checked by ICE (Synthego) to estimate the editing efficiency of gRNA, as summarized in Fig. 3B. Among these 9 clones, KO-1, KO-2, KO-5 and KO-9 were contaminated by wild type, although the contamination efficiency is lower than 3%. KO-3 and KO-6 were the mixture of different mutants. While KO-4, KO-7 and KO-8 were lack of 11 nucleotides with 100% efficiency. The PCR products of clones KO-1, KO-2, KO-5, KO-6 and KO-9 were cleaved into two fragments by T7 endonuclease assay as impure mutants (Fig. 3C). In contrast, the PCR products of KO-3, KO-4, KO-7 and KO-8 were not cleaved indicating that they might be pure clones. PtFucT1 overexpression and knockout mutants were also verified by qRT-PCR. The expression of PtFucT1 gene was significantly up-regulated in PtFucT1-OE mutants, while the expression was very low or not detected in PtFucT1-KO mutants (Additional file 1: Fig. S1). Additionally, the growth rate of these mutants was analyzed (Additional file 1: Fig. S2). The results showed that among different PtFucT1 overexpression and knockout mutants the growth rate did not have significant difference. Therefore, in this study, PtFucT1-OE1 and PtFucT1-KO8 mutants were selected for the subsequent experiments.

**Fig. 4:**
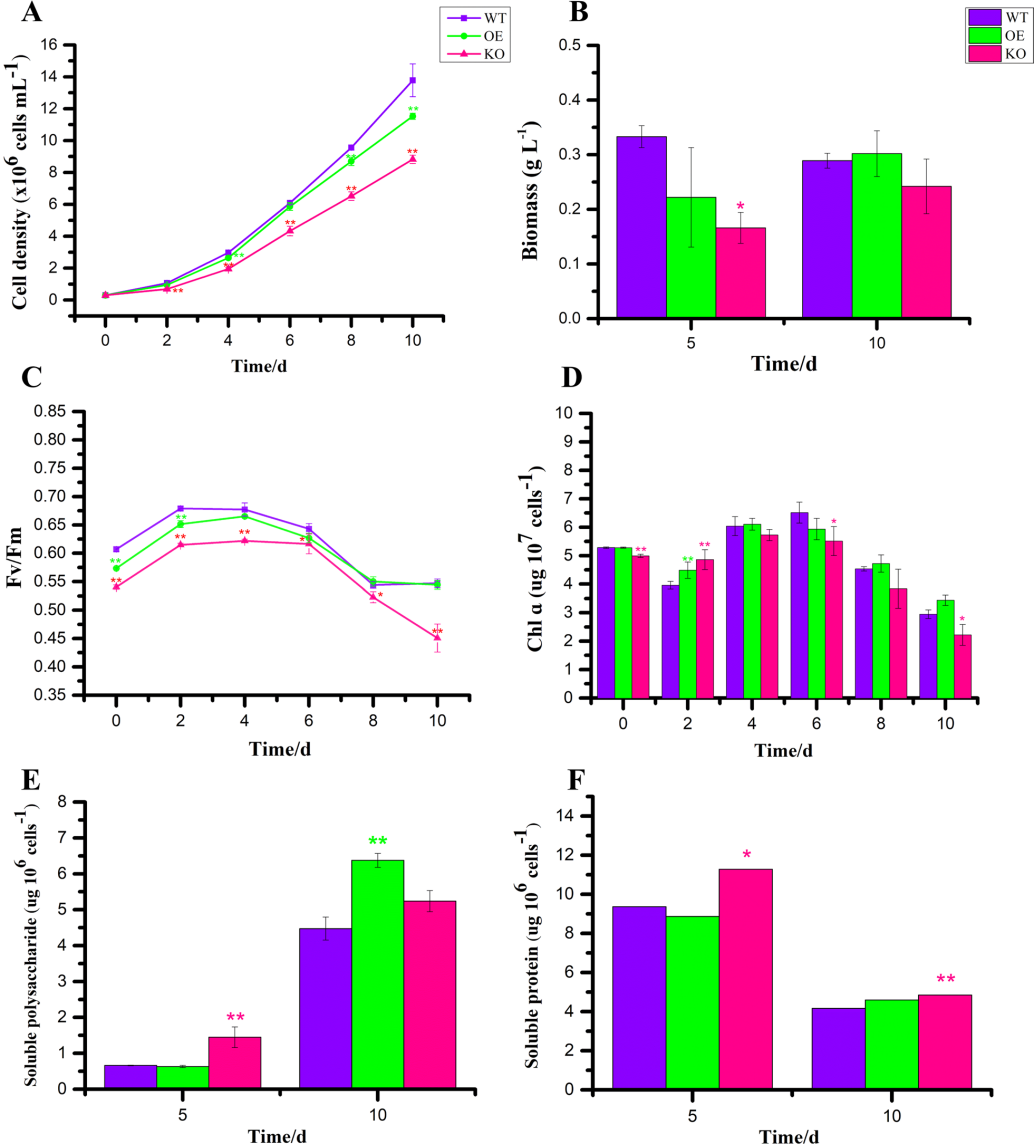
Physiological characteristics of PtFucT1 mutants. **A** The cell density of wild type, PtFucT1 overexpression mutant (OE) and PtFucT1 knockout mutant (KO); **B** biomass of wild type and PtFucT1 mutants under the 5th and 10th days; **C** and **D** the maximum effective quantum yield of photosystem II (Fv/Fm) and chlorophyll a content of wild type and PtFucT1 mutants; **E** and **F** contents of soluble polysaccharide and soluble protein in wild type and PtFucT1 mutants under the 5th and 10th days, respectively

After isolating and verifying the PtFucT1 knockout mutant, the physiological characteristics were analyzed (Fig. 4). Compared to wild type, overexpressing PtFucT1 gene (PtFucT1-OE) significantly decreased the cell density of *P. tricornutum* on the 4th, 8th, and 10th days (P < 0.01, Fig. 4A). The knockout of PtFucT1 gene (PtFucT1-KO) significantly reduced the cell density of *P. tricornutum* from the second day (P < 0.01). In addition, compared to wild type, the biomass did not have significant difference on the PtFucT1-OE, but it was significantly reduced on the 5th day of PtFucT1-KO (Fig. 4B).

Additionally, the Fv/Fm was measured for PtFucT1 mutants (Fig. 4C). The Fv/Fm of PtFucT1-OE on the first two days was significantly lower than that of wild type cells. In contrast, PtFucT1-KO Fv/Fm was significantly inhibited throughout the growth phase, and worse than that of PtFucT1-OE mutant. Chlorophyll a was decreased or significantly reduced in PtFucT1-KO compared to wild type (Fig. 4D). Particularly, the content of chlorophyll a was significantly increased on the 2nd day in PtFucT1-KO/OE mutants compared to that in wild type. Additionally, it was observed that the soluble polysaccharide content in the PtFucT1-KO/OE mutants was increased. However, the soluble protein was only remarkably accumulated in PtFucT1-KO mutant (Fig. 4).

### N-glycoproteomic characteristics of PtFucT1 mutants and N-glycan structures

#### General characteristics of N-glycoproteins in PtFucT1 mutants

**Fig. 5:**
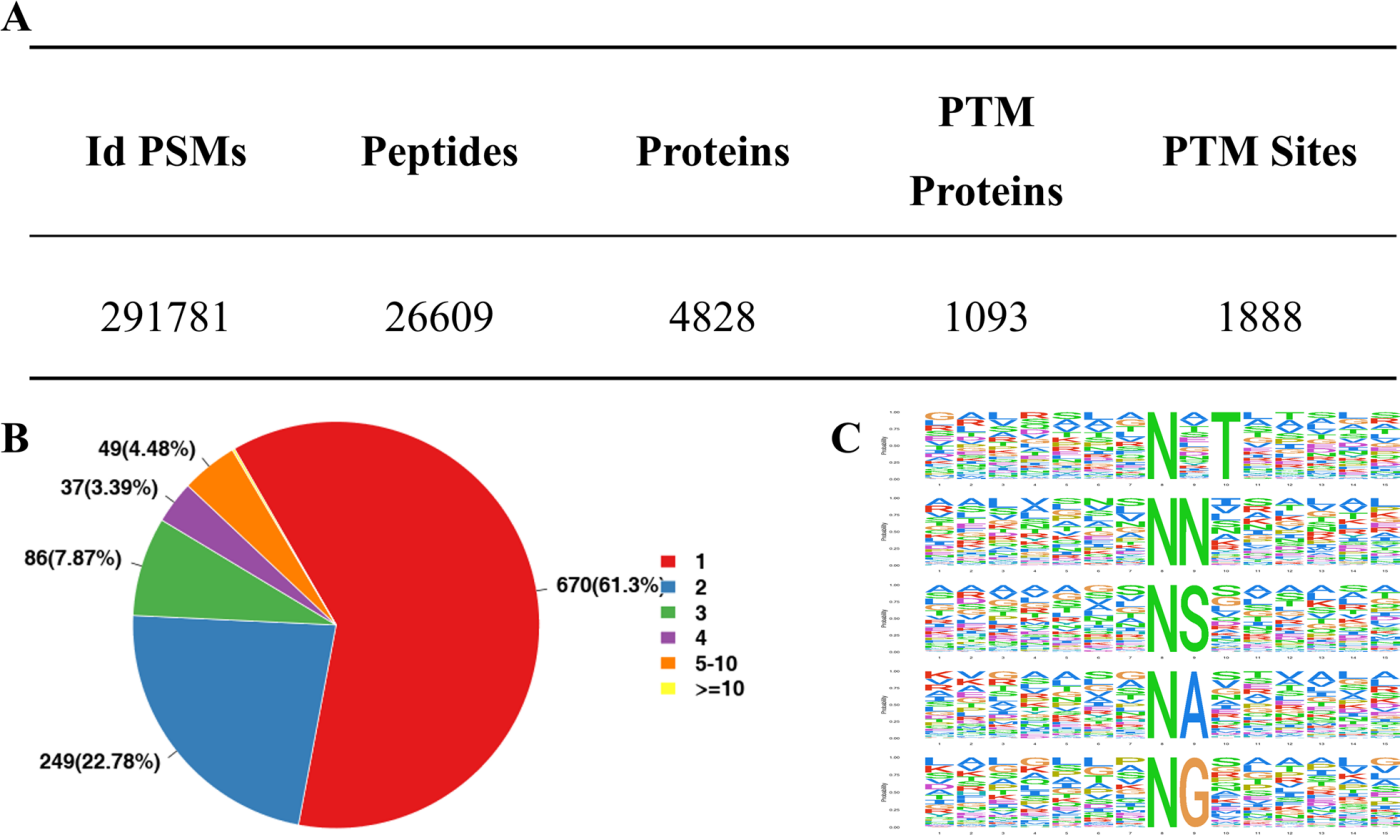
General characteristics of N-glycoproteins in PtFucT1 mutants. **A** The information of identified N-glycoproteins; Id PSMs, the number of identified spectrograms; Peptides, the number of identified peptides; Proteins, the number of identified proteins; PTM Proteins, the number of identified N-glycoproteins; PTM Sites, the number of post-translation modification (N-glycosylation modification) sites; **B** The number of N-glycosylation in proteins; **C** the conserved motifs of N-glycosylation modification sites

High throughput N-glycoproteome was carried out to establish the effects of PtFucT1 overexpression and knockout on the N-glycosylation modification of proteins (Fig. 5). A total of 291,781 spectrograms were identified from 26,609 peptides, which corresponded to 4828 proteins. Among these proteins, 1093 harbored 1888 N-glycosylated modification sites, of which 670 (61.3%) were only modified by one N-glycan (Fig. 5A). The number of N-glycoproteins decreased with an increase in the number of N-glycan modification sites (Fig. 5B). Eleven conserved N-glycosylated motifs were observed from all identified N-glycoproteins. The first five motifs with the stringent significance (Motif score > 10) were N_X_T (where x was an amino acid other than proline), NN, NS, NA and NG motifs (Fig. 5C).

#### Proteins with differentially N-glycosylation modification in PtFucT1 mutants

**Fig. 6:**
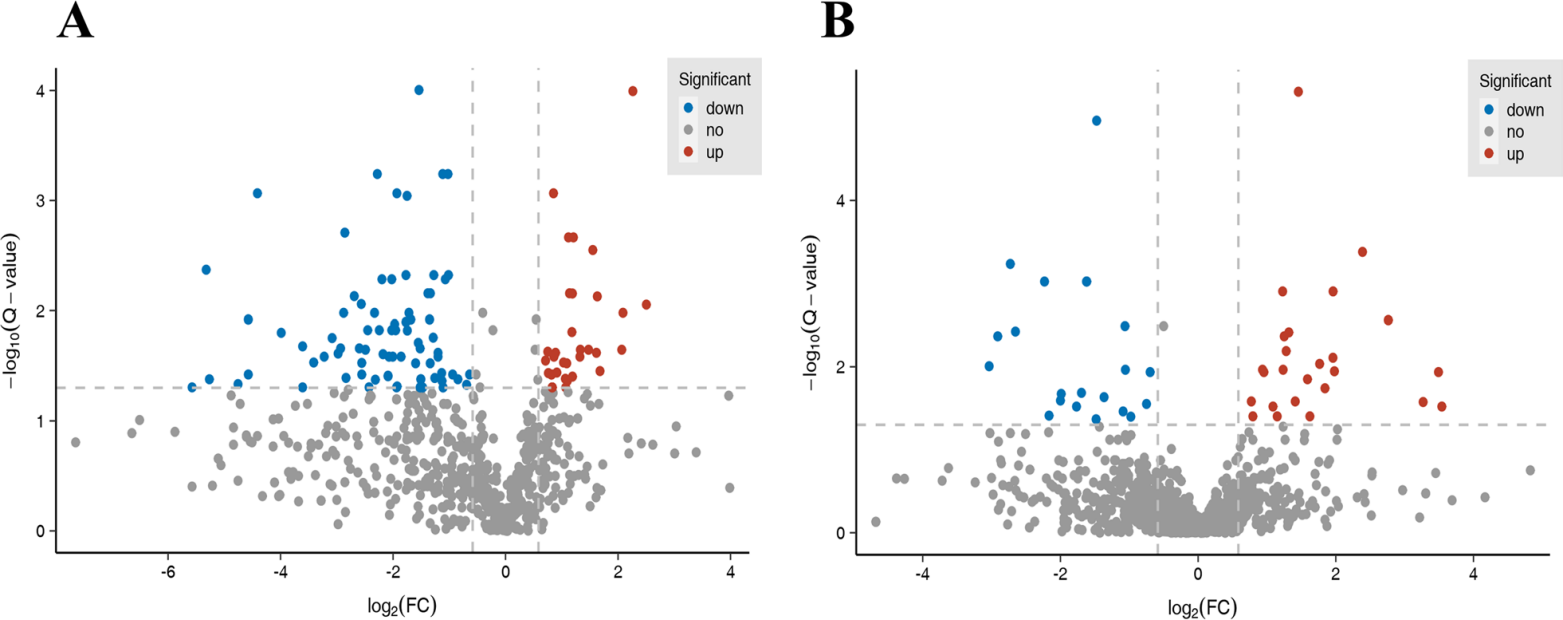
Differentially N-glycosylated proteins in PtFucT1 mutants. **A** and **B** the volcano plots of differentially N-glycosylation modification proteins (DGPs) in PtFucT1 overexpression and knockout mutants, respectively

Furthermore, differentially N-glycosylated proteins (DGPs) were characterized (q-value ≤ 0.05) with the fold change ≥ 1.5 classified as up-regulated N-glycosylation, while a fold change ≤ 0.67 classified as down-regulated N-glycosylation. A total of 111 sites from 97 N-glycoproteins were differentially N-glycosylated in the PtFucT1-OE. Compared to the wild type, 35 N-glycosylated sites from 30 N-glycoproteins were up-regulated, and 76 N-glycosylated sites from 67 N-glycoproteins were down-regulated in the PtFucT1-OE mutant (Fig. 6A). In PtFucT1-KO, 46 sites from 40 N-glycoproteins were differentially N-glycosylated, resulting in the up-regulation of 25 N-glycosylated sites from 20 N-glycoproteins and down-regulation of 21 N-glycosylated sites from 20 N-glycoproteins (Fig. 6B). The DGPs showed that 18 N-glycoproteins were common between the PtFucT1-OE and PtFucT1-KO mutants, 79 were unique to the PtFucT1-OE mutant, and 22 were unique to the PtFucT1-KO mutant. The predicted proteins in PtFucT1-OE and PtFucT1-KO mutants were 64 (66%) and 28 (70%) N-glycoproteins, respectively.

Annotations were carried out using different database, including GO, COG and KEGG, to evaluate the DGPs at the functional level (Additional file 1: Fig. S3–S5). The GO annotation showed that DGPs in the biological process, cellular component, and molecular function classes in PtFucT1-OE mutant were mainly involved in cellular and metabolic processes [e.g. protein homeostasis related B7FW63 (protein disulfide isomerase activity)], cellular anatomical entities [e.g. photosynthesis related A0T0M6 (psaL)] and protein-containing complexes [e.g. photosynthesis related A0T096 (psbC)], and catalytic and binding activities [e.g. protein homeostasis related B7FP06 (proteasome subunit alpha type)], respectively. Similar results were also observed in PtFucT1-KO mutant (Additional file 1: Fig. S3). The COG annotation showed that the most DGPs in PtFucT1-OE mutant were linked to post-translational modification [e.g. protein homeostasis related A0T0F2 (ATP-dependent Zn proteases)], protein conversion and chaperones [e.g. protein homeostasis related B7FQ72 (HSP60)], energy production and conversion, lipid transport and metabolism. In addition to these processes, the main DGPs in PtFucT1-KO mutant were further linked to carbohydrate transport and metabolism (Additional file 1: Fig. S4). Annotating in the KEGG pathway in PtFucT1-OE and PtFucT1-KO mutants revealed that most DGPs were belonged to the metabolism class, particularly the metabolic pathways, microbial metabolism in diverse environments, and biosynthesis of secondary metabolites (Additional file 1: Fig. S5). Moreover, it was predicted that most of these DGPs were targeted to chloroplast, cytoplasm, nucleus, and plasma membrane in two mutants (Additional file 1: Fig. S6). Biological process, cellular component, molecular function, and KEGG enrichment (based on GO and KEGG database) revealed that most of the DGPs were associated with a negative regulation of cellular metabolism and cellular aldehyde metabolic process, chloroplast and microtubule, ligase activity and forming carbon–sulfur bond, and photosynthesis in PtFucT1-OE mutant, respectively. While in PtFucT1-KO mutant, most DGPs were associated with carbohydrate metabolism in biological process enrichment, cell wall and external encapsulating structure in cellular component enrichment, carbon–carbon lyase activity in molecular function enrichment, and glyoxylate and dicarboxylate metabolism in KEGG enrichment (data not shown).

To analyze the relationship between the physiological characteristics and N-glycoproteins, some proteins with differentially N-glycosylation modifications were identified (Table 1). In PtFucT1-OE mutant, the N-glycosylation modifications of the two proteins (tubulin (TUB)-α and β chains) function on the cytoskeleton were down-regulated. The modification of Photosystem I reaction center subunit XI (PsaL) involved in photosynthesis was up-regulated. In addition, three down-regulated N-glycoproteins (Glucose-6-Phosphate Isomerase (GPI_1), Citrate synthase (Cts) and Glyceraldehyde-3-phosphate dehydrogenase (GapC2a)) in carbon metabolism were also identified from PtFucT1-OE mutant (Table 1). The N-glycosylation modifications of the three proteins (Protein fucoxanthin chlorophyll a/c protein (Lhcr6), Ribulose bisphosphate carboxylase large chain (rbcL) and Violaxanthin de-epoxidase (Vde)) linked to photosynthesis processes in PtFucT1-KO mutant were up-regulated. The N-glycosylation modifications of Carbohydrate kinase (CarK) and Fructose-bisphosphate aldolase (FbaC2) linked to carbon metabolism were up-regulated, while the N-glycosylation of Isocitrate lyase (Isol) was down-regulated. The N-glycosylation modification of Agmase and glutamine synthesis (GS) from the process of nitrogen metabolism was down-regulated in PtFucT1-KO mutant (Table 1).

**Table 1:** Partially proteins with differentially N-glycosylation modification in PtFucT1 mutants

	Protein accession	Gene name	Site	N-gly	Description
PtFucT1 overexpression mutant					
Cytoskeleton	B7G0C3	TUB-α	226	Down	Tubulin alpha chain
	B5Y3W7	TUB-β	14	Down	Tubulin beta chain
Photosynthesis	A0T0M6	psaL	112	Up	Photosystem I reaction center subunit XI
	A0T0F2	ftsH	615	Down	ATP-dependent zinc metalloprotease FtsH
	B7G503	Lhcr14	173	Down	Protein fucoxanthin chlorophyll a/c protein
	B5Y578	PetJ	103	Down	Cytochrome c-553
	A0T096	psbC	380	Down	Photosystem II CP43 reaction center protein
	A0T096	psbC	325	Down	Photosystem II CP43 reaction center protein
	B7FZ96	PsbO	68	Down	Oxygen-evolving enhancer protein 1
	Q9TK52	rbcL	313	Down	Ribulose bisphosphate carboxylase large chain
	A0T0G9	psbA	266	Down	Photosystem II protein D1
	A0T0G9	psbA	267	Down	Photosystem II protein D1
	Q9TK52	rbcL	290	Down	Ribulose bisphosphate carboxylase large chain
	B7G532	Atp1	189	Down	ATP synthase subunit alpha
	B5Y3C9	Cytb6f	74	Down	Cytochrome B6-F complex
Carbon metabolism	B7GDK9	GPI_1	472	Down	Glucose-6-phosphate isomerase
	B7G9P5	Cts	163	Down	Citrate synthase (TCA)
	B7G6K6	GapC2a	205	Down	Glyceraldehyde-3-phosphate dehydrogenase
PtFucT1 knockout mutant					
Photosynthesis	B7G4U8	Lhcr6	146	Up	Protein fucoxanthin chlorophyll a/c protein
	Q9TK52	rbcL	442	Up	Ribulose bisphosphate carboxylase large chain
	Q9TK52	rbcL	37	Up	Ribulose bisphosphate carboxylase large chain
	B7FUR6	Vde	397	Up	Violaxanthin deepoxidase
Carbon metabolism	B5Y5B6	CarK	338	Up	Carbohydrate kinase
	B7G9G9	FbaC2	384	Up	Fructose-bisphosphate aldolase
	B7G518	Isol	342	Down	Isocitrate lyase
Nitrogen metabolism	B7GCN5	Agmase	284	Down	Agmatinase
	B7G5A1	GS	385	Down	Glutamine synthetase

Note: *N-gly,* N-glycosylation of the protein

#### N-glycan structures of proteins in PtFucT1-KO mutant

**Fig. 7:**
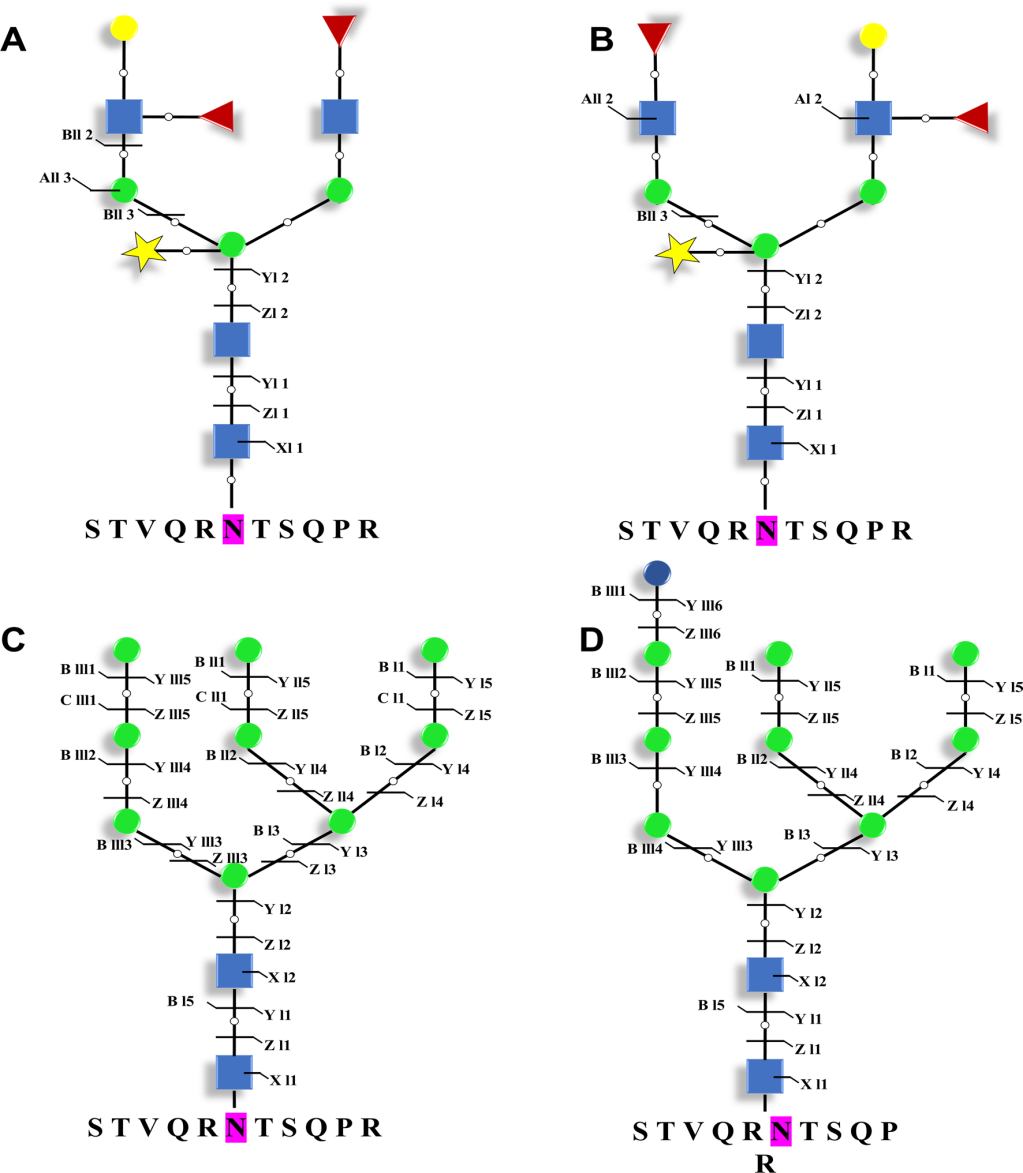
N-glycan structures of copper transporter (B7G8E0) from wild type (**A** and **B**) and PtFucT1-KO mutant (**C** and **D**). Yellow circle, galactose; yellow star, Xylose; red triangle, fucose; green circle, mannose; blue circle, glucose; blue square, N-acetylglucosamine

To further evaluate the effects of PtFucT1-KO on N-glycan structures, N-glycomics study was carried out. A total of 36 different N-glycoproteins were identified from the N-glycomics. About 88 N-glycopeptides were identified from the wild type while 83 N-glycopeptides were identified from PtFucT1-KO mutant (Additional file 3: Table S4 and S5). These N-glycopeptides were mainly classified into four different types, N-glycopeptides modified by core α-1,3-fucose residue (α-1,3-fucose) (14, 8.19%), N-glycopeptides modified by non-core fucose residue (α-1,4-fucose) (75, 43.86%), N-glycopeptides modified by non-core and/ or core fucose residues (7, 4.09%), and N-glycopeptides without fucose modification (75, 43.85%). It was shown that 75 N-glycopeptides from 28 different N-glycoproteins were modified by non-core α-1,4-fucose residues in wild type, but not modified by fucose residues in PtFucT1-KO mutant. Interestingly, the structures of N-glycan in wild type were mainly complex type N-glycans with four GlcNAc, two fucose and one xylose residues, while the structures in PtFucT1-KO mutant were composed of mannose type N-glycans with 4–10 mannose residues, and without fucose and xylose residues. Here, taking a protein (B7G8E0) for example, it was annotated to be involved in copper transport. The N-glycosylation modification of this protein was happened on the 167th amino acid. The analysis of N-glycan structure showed that this site might be modified by two different N-glycan structures, Gal_1_Xyl_1_FuT_2_GlcNAc_2_Man_3_GlcNAc_2_ (Gal, galactose; Xyl, Xylose; FuT, fucose; Man, mannose; GlcNAc, N-acetylglucosamine), depicted in Fig. 7A and B. However, two diverse N-glycan structures of this copper transport protein were identified in PtFucT1-KO mutant, Man_9_GlcNAc_2_ and Glc_1_Man_9_GlcNAc_2_ (Glc, glucose) (Fig. 7C and D). In addition to this protein, the N-glycan structures of B7G280 without known function were also shown in Additional file 1: Fig. S7.

Additionally, compared to 7 core α-1,3-fucose modified N-glycopeptides in wild type, core fucose residues were also found from the same 7 N-glycopeptides in PtFucT1-KO mutant. These 7 glycopeptides were corresponding to three different proteins (B7GBU2, B7GEM5 and B7GEB5). Among them, B7GBU2 and B7GEM5 were annotated to be predicted proteins without known function, while B7GEB5 was annotated to be putative Acetyl-CoA carboxylase via the server of SWISS-MODEL. The N-glycan structures of putative Acetyl-CoA carboxylase Xyl_1_FuT_1_Man_5_GlcNAc_2_ from wild type and PtFucT1-KO mutant were depicted (Additional file 1: Fig. S8).

**Fig. 8:**
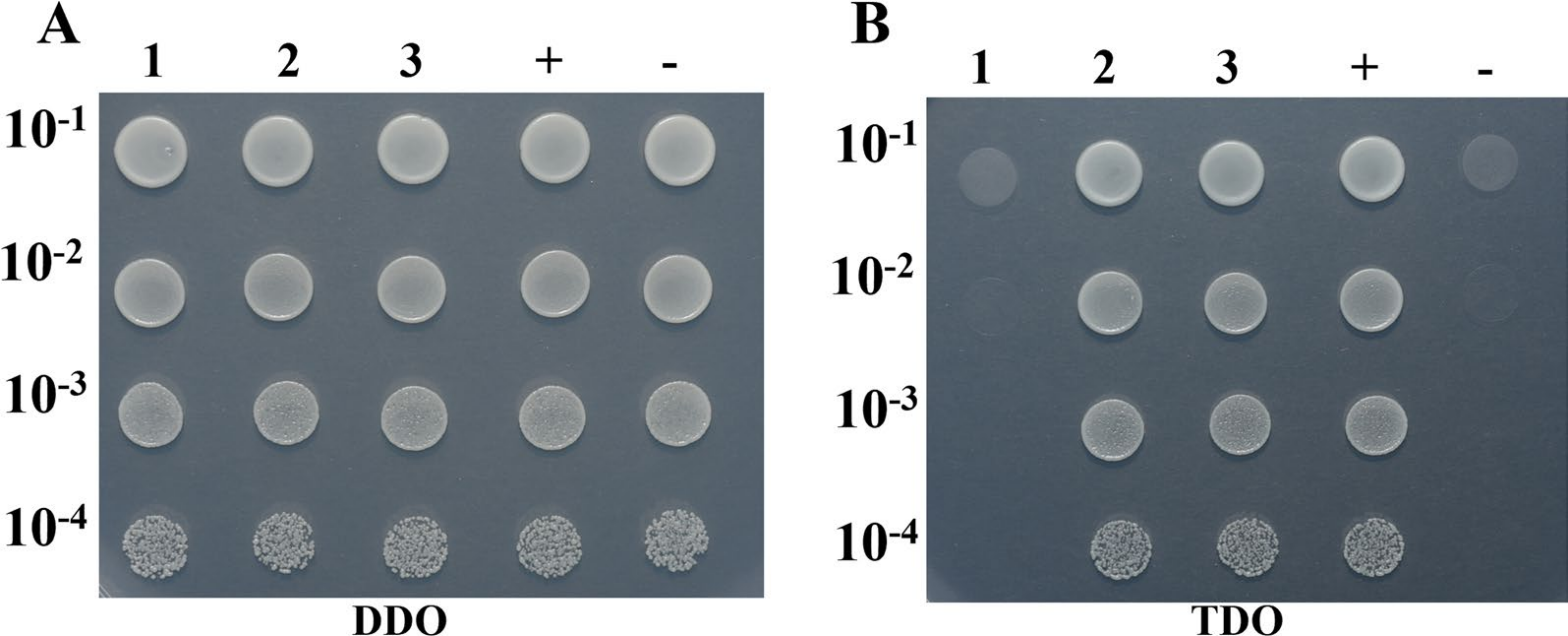
The analysis of PtFucT1 and PtGnT1 interaction. **1** pBT3-N- FucT1 and pPR3-N (self-activation); **2** pBT3-N- FucT1 and pOst1-NubI (functional verification); **3** pBT3-N-FucT1 and pPR3-N- GnT1 (experimental group); + : pTSU2-APP and pNubG-Fe65 (positive control); -: pTSU2-APP and PPR3-N (negative control). SD: synthetical defined medium. DDO: SD/-Leu/-Trp nutrient deficient medium. TDO: SD/-Leu/-Trp/-His nutrient deficient medium

To preliminary analyze why the knock-out of PtFucT1 gene change the complex N-glycans to mannose type N-glycans, a yeast two-hybrid experiment was carried out between PtFucT1 and PtGnT1. It is shown in Fig. 8. The results showed that the yeast could growth on SD/-Leu/-Trp/-His medium (Fig. 8B-3).

## Discussions

Pathways of protein N-glycosylation modification were previously proposed in some microalgae, such as *C. reinhardtii*, *Chlorella vulgaris*, and *P. tricornutum* [4, 48, 49]. The putative glycosyltransferases (GTs) and glycosidases (GSs) that participated in the ER and Golgi N-glycosylation pathway were identified from different microalgae [4, 50]. The N-glycosylation pathways of protein in microalgae were classified into GnTI-dependent and GnTI-independent pathways. However, the complete and precise pathway in microalgae remain largely unknown. A few studies explored the pathways of the genes involved in protein N-glycosylation in microalgae [4]. In *C. reinhardtii*, a study on the functions of GnTI, mannosidase 1A (Man1A), xylosyltransferases (XylT1A (XTA), XylT1B (XTB)) and fucosyltransferase (FucT) revealed that the N-glycosylation process followed the GnT I-independent pathway [51]. The Man1A insertion mutant affected methylation of mannoses and the addition of terminal xylose while the absence of XylT1A resulted in shorter N-glycan structure compared to that of wild type [52]. XTA was responsible for the core β-1,2-xylose modification and XTB the β-1,4-xylose modification on the linear branch of the N-glycan and partly the core β-1,2-xylose modification [53]. Moreover, knocking down FucT affected Man1A-depedent trimming and fucose transfer on N-glycan, while the knockdown of both XylTs and FucT lead to the formation of N-glycans with strongly diminished core modifications [15]. The functions of several genes were also studied in *P. tricornutum*. N-acetylglucosaminyltransferase I (PtGnTI) could restore the maturation of complex type N-glycans in the Chinese Hamster Ovary (CHO) Lec1 mutant lacking endogenic GnT I, thus demonstrating the functional activity of the diatom N-acetylglucosaminyltransferase [3]. A putative GDP-L-fucose transporter (PtGFT) was able to rescue the fucosylation of proteins in the CHO-gmt5 mutant cell line, suggesting the potential transporter function of PtGFT in *P. tricornutum* [20]. A preliminary study of PtFucT1 (54,599) function via α-1,3-fucose antibody was already carried out in *P. tricornutum*, and it showed that the overexpression of FucT increased the N-glycans bearing α-1,3-fucose epitope [20].

Among 7 glycosyltransferase (GT) families, the GT37 family are certificated to be involved in the fucosylation of plant-specific organelles, such as cell wall, while GT10 family contained α-1,3/4-FucTs for N-glycans in plants [9]. The three PtFucTs were clustered in GT10 family, indicating their putative functions as α-1,3/4-FucTs on N-glycans. Among the five conserved motifs of plant FucTs from GT10 family, three PtFucTs contained the highly conserved Motif IV and Motif V. Two of these potentially functional motifs were also found in invertebrates [54] and plant α-1,3-FucT via Pfam analysis [55]. It was reported that motif IV can interact with the donor substrate GDP-fucose for the fucosyltransferase activity [20, 55]. The CXXC motif (Motif V) was located at the C-terminus of the three PtFucTs, which is often involved in the formation of disulfide bonds in plant α-1,3-FucT [48]. The previous paper showed that the three PtFucTs contained conserved motif III to motif V [20]. However, because motif III was not so highly conserved, it was not thought to be conserved motif in this study.

The subcellular localization of a protein is important for its function in cell. Until now, GDP-fucose transporter (PtGFT) was located to the Golgi apparatus in the CHO-gmt5 mutant cell line, PtGnTI and PtFucT1 were verified to be medial/ trans-Golgi localization via transmission electron microscope coupled to immune-gold labeling in *P. tricornutum* [20]. The medial/ trans-Golgi located PtFucT1 was further confirmed in our study by eGFP fusion expression and co-expression with Golgi marker PtXylT/PtVps29-mRFP via confocal laser scanning microscopy [21]. Additionally, owing to the plastid stroma-like fluorescence [21], PtFucT2 and PtFucT3 lacking plastid targeting information were localized to the plastid stroma. However, it is still unknown how to explain the plastid stroma localization of PtFucT2 and PtFucT3. It was proposed that the plastid of the *Chlamydomonas* cells does not contain the N-glycosylation machinery [56], indicating that PtFucT2 and PtFucT3 might not participate in the N-glycosylation modification of protein. Therefore, it is interesting to study the function of Golgi-located PtFucT1 during the protein N-glycosylation pathway.

Subsequently, PtFucT1-OE mutant and the knockout mutant of PtFucT1 (PtFucT1-KO) lacking two main motifs (Motif IV and V) were analyzed in physiological and N-glycoproteomic levels. Tubulin proteins are an important constituent of microtubules that build the cytoskeleton [57] and regulate cell elongation and growth [58]. Therefore, the significant decrease of cell density was probably due to the down-regulated N-glycosylation of tubulin alpha and beta chains in PtFucT1-OE mutant compared to the wild type cells. PsbA affected primary photochemistry, such as chlorophylls and the transfer of electron in Photosynthesis II [59]. Gene rbcL participated in the CO_2_ fixation and regulated the plant biomass [60]. Lhcr14 encoding the fucoxanthin chlorophyll a/c protein is a unique light-harvesting apparatus in diatom [61]. Besides, ftsH is important for the growth of leaf and the development of chloroplast [62]. All these relevant proteins regulated the photosynthesis efficiency in PtFucT1-OE mutant. Therefore, the reduced Fv/Fm in PtFucT1-OE mutant might be related with the up-regulated N-glycosylation of psaL and down-regulated N-glycosylation of the other nine photosynthesis relevant proteins (ftsH, Lhcr14, PetJ, PsbC, PsbO, rbcL, PsbA, Atp1 and Cytb6f). The increased soluble polysaccharide in PtFucT1-OE mutant was probably due to the down-regulation of the N-glycosylation of GPI_1, Cts and GapC2a. It was already known that these three key enzymes were important during the processes of glycolysis or tricarboxylic acid cycle as they regulated carbon metabolism [63]. The DGPs involved in the cytoskeleton were not identified in PtFucT1-KO mutant. However, seven DGPs related with photosynthesis, carbon and nitrogen metabolisms were found in PtFucT1-KO mutant. Especially, glutamine synthetase is a key enzyme for the assimilation of nitrogen [26]. The up- or down-regulated N-glycosylation of these proteins might change the proteins’ structure and subsequent function, leading to various physiological phenotypes in PtFucT1-KO mutant.

**Fig. 9:**
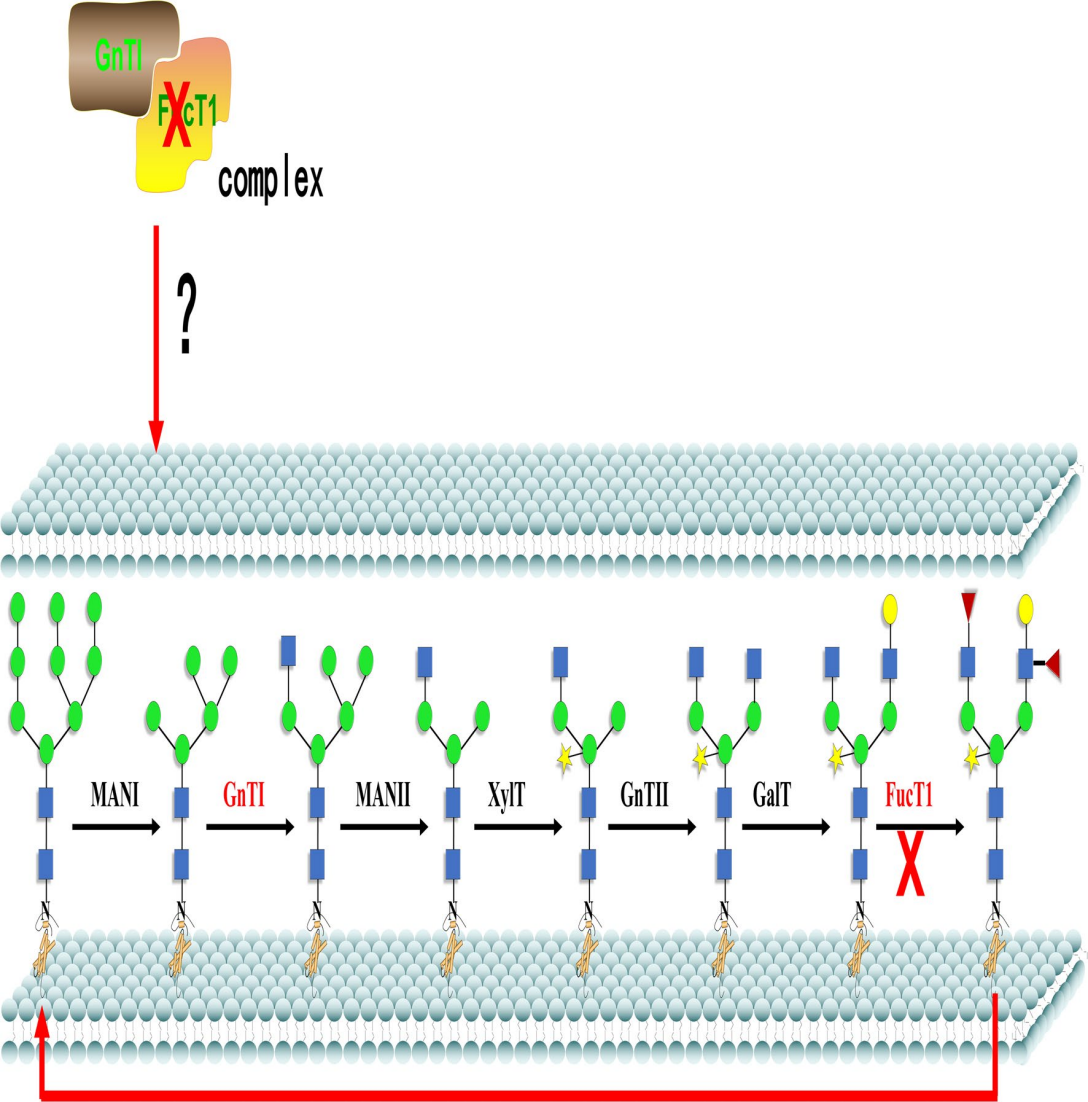
The proposed working model of PtFucT1. Man I, α-mannosidase I; GnTI, N-acetylglucosaminyltransferase I; Man II, α-mannosidase II; XylT, xylosyltansferase; GnTII, N-acetylglucosaminyltransferase II; GalT, galactosyltransferase

N-glycan structures are important for the protein folding, structure stability, and function [5]. However, the N-glycans of the DGPs discussed in this study were not identified in the N-glycomics. Although previous studies showed that the overexpression of PtFucT1 increased the core fucose modified N-glycoproteins [20], the knockout of PtFucT1 inactivated the α-1,4-fucose modification but not the core α-1,3-fucose modification of N-glycans in the present study. This is consistent with the functional prediction, which showed that PtFucT1 is a non-core fucosyltransferase. The change of complex type N-glycans in wild type to mannose type in PtFucT1-KO mutant indicated that the knockout of PtFucT1 not only inhibited the functions of α-1,4-fucosyltransferase, but also the activity of PtGnTI. This was certificated by the yeast two-hybrid experiment, where showed that PtFucT1 and PtGnTI can interact. PtGnTI is responsible for the synthesis of complex N-glycans [3]. The knock-out of PtFucT1 inhibited the activity of PtGnTI and then changed the N-glycan structure. The proposed working model of PtFucT1 was shown in Fig. 9.

Interestingly, the core α-1,3-fucose residues of N-glycans were both observed in wild type and PtFucT1-KO mutant, suggesting the existence of core fucosyltransferase in *P. tricornutum*. For example, Acetyl-CoA carboxylase (ACC, B7GEB5) harboring core α-1,3-fucose residue was observed in both the wild type and PtFucT1-KO mutant. ACC is a key enzyme responsible for the catalysis of carboxylation of acetyl-CoA into malonyl-CoA in plastids [64]. However, it is still unknown which protein is responsible for the core fucose modification. The cause of the absence of fucose residue in 13 N-glycopeptides (7.6%) in the wild type while they have fucose residue in the PtFucT1-KO mutant is unknown. We speculate that the difference could be due to the remaining first domain (97–229AA) in PtFucT1-KO mutant or failure to knockout the first domain of PtFucT1 in *P. tricornutum*. In addition to the reported main mannose type N-glycans (5–9 mannose residues) in *P. tricornutum* [3, 65], large amount of complex N-glycans with GlcNAc, fucose and xylose residues were identified in this study. Diatoms may initially synthesized mannose type N-glycans, and then the complex type N-glycans [65], with the Glc_2_Man_9_GlcNAc_2_ structure being the lipid-linked oligosaccharide in *P. tricornutum* [66]. Although the main N-glycan structure and lipid-linked oligosaccharide were reported, to our knowledge, this is the first time the total N-glycoproteins and their corresponding precise N-glycans were analyzed using N-glycoproteomic and N-glycomic levels in *P. tricornutum*. This provided critical data for further functional study of these N-glycoproteins and the mechanism of protein N-glycosylation modification.

## Supplementary Information

**Additional file 1:** **Figure S1.** The relative expression of PtFucT1 gene in PtFucT1 mutants. **Figure S2.** The cell density of wild type and PtFucT1 mutants. **Figure S3.** Gene Ontology (GO) functional annotation of differentially N-glycosylation modification proteins in PtFucT1 mutants. **Figure S4.** Clusters of Orthologous Groups of protein (COG) functional annotation of differentially N-glycosylation modification proteins in PtFucT1 mutants. **Figure S5.** Kyoto Encyclopedia of Genes and Genomes (KEGG) functional annotation of differentially N-glycosylation modification proteins in PtFucT1 mutants. **Figure S6.** Subcellular localization of differentially N-glycosylation modification proteins in PtFucT1-OE (A) and PtFucT1-KO (B) mutants. **Figure S7.** N-glycan structures on the 19th amino acid of the predicted protein (B7G280) from wild type (A and B) and PtFucT1-KO mutant (C and D). **Figure S8.** N-glycan structures of putative Acetyl-CoA carboxylase (B7GEB5) from wild type (A) and PtFucT1-KO mutant (B).

**Additional file 2:** **Table S1.** Primers used in this paper. **Table S2.** Differentially N-glycosylated proteins in PtFucT1 overexpression mutant. **Table S3.** Differentially N-glycosylated proteins in PtFucT1 knockout mutant.

**Additional file 3:** **Table S4.** N-glycan structures of Glycoproteins in wild type. **Table S5.** N-glycan structures of Glycoproteins in PtFucT1-KO.

## Data Availability

The data that support the findings of this study are available from the corresponding author upon reasonable request.
