# Supplementary material for: Golgi fucosyltransferase 1 reveals its important role in α-1,4-fucose modification of N-glycan in CRISPR/Cas9 diatom Phaeodactylum tricornutum
Source: Microb Cell Fact. 2023 Jan 7;22:6. doi: 10.1186/s12934-022-02000-2 (PMC9826595; doi:10.1186/s12934-022-02000-2)
Supplement: Supplementary file 2 — Additional file 2: Table S1. Primers used in this paper. Table S2. Differentially N-glycosylated proteins in PtFucT1 overexpression mutant. Table S3. Differentially N-glycosylated proteins in PtFucT1 knockout mutant. [file 12934_2022_2000_MOESM2_ESM.docx]

Golgi fucosyltransferase 1 reveals its role in α-1,4-fucose modification of N-glycan in Diatom *Phaeodactylum tricornutum*

Xihui Xie, Jianchao Yang, Hong Du, Jichen Chen, Edmond Sanganyado, Yangmin Gong, Wanna Wang, Weizhou Chen, Zhengyi Liu, Xiaojuan Liu

Table S1. Primers used in this paper

Table S2. Differentially N-glycosylated proteins in PtFucT1 overexpression mutant

Table S3. Differentially N-glycosylated proteins in PtFucT1 knockout mutant

Table S1. Primers used in this paper

| Gene name | Primers（5’-3’） |
| --- | --- |
| PtFucT1-F | gactaattcgagctcggtaccATGTCACTTCGCAAGCGTGG |
| PtFucT1-R | ttgctcaccatCGGATCGAACTTCCACGAGG |
| PtFucT1-eGFP-F | ttcgatccgATGGTGAGCAAGGGCGAGG |
| PtFucT1-eGFP-R | tctagaggatccccgggtaccTTACTTGTACAGCTCGTCCATGCC |
| PtFucT2-F | cacttgtgcgaacggaattcATGGTATCAATCCGCAACCCCA |
| PtFucT2-R | gcccttgctcaccatTGACGATACGTAAAATAACTCG |
| PtFucT2-eGFP-F | ttttacgtatcgtcaATGGTGAGCAAGGGCGAGGAGC |
| PtFucT2-eGFP-R | aggatccccgggtaccTTACTTGTACAGCTCGTCCATG |
| PtFucT3-F | cacttgtgcgaacggaattcATGATTCGCATACCATACGCGG |
| PtFucT3-R | gcccttgctcaccatCACGAAGGTTTCCACAGGATTC |
| PtFucT3-eGFP-F | gtggaaaccttcgtgATGGTGAGCAAGGGCGAGGAGC |
| PtFucT3-eGFP-R | aggatccccgggtaccTTACTTGTACAGCTCGTCCATG |
| PtFucT1-836-F  (gRNA) | TCGAGTTGCAGTTAGACACCACCC |
| PtFucT1-836-R  (gRNA) | AAACGGGTGGTGTCTAACTGCAAC |
| PtFucT1-OE-F  (qRT-PCR) | CGACGAAGCCGAATTTCTTC |
| PtFucT1-OE-R  (qRT-PCR) | GGATTGAGTTGTAGGGTTGG |
| PtFucT1-KO-F  (qRT-PCR) | TGGGTGGTGTCTAACTGCAA |
| PtFucT1-KO-R  (qRT-PCR) | CGTTGCAGAAGGTGTTTTCA |
| 30S ribosomal protein subunit-F (RPS) | CGAAGTCAACCAGGAAACCAA |
| 30S ribosomal protein subunit-R (RPS) | GTGCAAGAGACCGGACATACC |

Notes: PtFucT1-F, the forward primer for PtFucT1 in pPha-NR-PtFucT1-eGFP plasmid; PtFucT1-R, the reverse primer for PtFucT1 in pPha-NR-PtFucT1-eGFP plasmid; PtFucT1-eGFP-F, the forward primer for eGFP in pPha-NR-PtFucT1-eGFP plasmid; PtFucT1-eGFP-R, the reverse primer for eGFP in pPha-NR-PtFucT1-eGFP plasmid; The notes for the primers of PtFucT2-3 are the same.

Table S2. Differentially N-glycosylated proteins in PtFucT1 overexpression mutant

| **Gene** | **Protein**  **Accession** | **Position** | **Fold Change** | **State** | **Description** |
| --- | --- | --- | --- | --- | --- |
| PHATRDRAFT_46010 | B7FZH7 | 63 | 10.723 | up | Predicted protein |
| PHATR_33351 | B5Y520 | 283 | 5.672 | up | Predicted protein |
| PHATRDRAFT_51092 | B7G5A1 | 385 | 4.802 | up | Glutamine synthetase |
| PHATRDRAFT_18319 | B7FT14 | 366 | 4.251 | up | Adenosylhomocysteinase |
| PHATRDRAFT_45403 | B7FXK5 | 153 | 4.187 | up | Predicted protein |
| PHATRDRAFT_8770 | B7FR62 | 138 | 3.203 | up | Predicted protein |
| PHATRDRAFT_45403 | B7FXK5 | 145 | 3.098 | up | Predicted protein |
| PHATR_46710 | B5Y3J4 | 618 | 3.064 | up | Predicted protein |
| PHATRDRAFT_12799 | B7G0J8 | 51 | 2.931 | up | Prohibitin |
| PHATRDRAFT_45088 | B7FWF2 | 409 | 2.782 | up | Predicted protein |
| PHATRDRAFT_47510 | B7G3M5 | 162 | 2.514 | up | Predicted protein |
| PHATRDRAFT_23164 | B7GAC9 | 309 | 2.5 | up | Peptidylprolyl isomerase |
| PHATRDRAFT_49091 | B7G9G6 | 64 | 2.308 | up | Predicted protein |
| PHATRDRAFT_44868 | B7FVX8 | 75 | 2.281 | up | Predicted protein |
| PHATRDRAFT_2808 | B7FW63 | 169 | 2.279 | up | Predicted protein |
| PHATRDRAFT_55162 | B7GDF5 | 61 | 2.265 | up | Predicted protein |
| PHATRDRAFT_bd1566 | B7S3M4 | 192 | 2.199 | up | Predicted protein |
| PHATRDRAFT_50500 | B7GEA6 | 162 | 2.171 | up | Predicted protein |
| PHATRDRAFT_45403 | B7FXK5 | 57 | 2.14 | up | Predicted protein |
| PHATRDRAFT_50268 | B7GDI3 | 341 | 2.125 | up | Predicted protein |
| psaL | A0T0M6 | 112 | 2.099 | up | Photosystem I reaction center subunit XI |
| Sec4 | B7G9L9 | 197 | 2.089 | up | Predicted protein |
| PHATRDRAFT_27757 | B7G0L4 | 213 | 2.043 | up | Predicted protein |
| PHATRDRAFT_55162 | B7GDF5 | 170 | 1.882 | up | Predicted protein |
| PHATRDRAFT_48536 | B7G7L3 | 356 | 1.852 | up | Predicted protein |
| PHATRDRAFT_42498 | B7FRH9 | 572 | 1.815 | up | Predicted protein |
| PHATRDRAFT_45403 | B7FXK5 | 149 | 1.805 | up | Predicted protein |
| PHATRDRAFT_45250 | B7FWZ9 | 124 | 1.777 | up | Predicted protein |
| PHATRDRAFT_22974 | B7G9F0 | 544 | 1.777 | up | Acetyl-coenzyme A synthetase |
| PHATRDRAFT_bd645 | B7S3P0 | 601 | 1.777 | up | Acetyl-coenzyme A synthetase |
| PHATR_46768 | B5Y3Q5 | 243 | 1.761 | up | Protein xylosyltransferase |
| PHATRDRAFT_8975 | B7FQ11 | 52 | 1.761 | up | Glycerol-3-phosphate dehydrogenase [NAD(+)] |
| PHATRDRAFT_42545 | B7FRN8 | 127 | 1.695 | up | Predicted protein |
| PHATRDRAFT_55162 | B7GDF5 | 115 | 1.682 | up | Predicted protein |
| PHATRDRAFT_23497 | B7GBX2 | 229 | 1.637 | up | Predicted protein |
| GLXI | B7G085 | 159 | 0.642 | down | Lactyolglutathione lyase |
| PHATRDRAFT_21548 | B7G341 | 435 | 0.619 | down | Predicted protein |
| PHATRDRAFT_51088 | B7G518 | 342 | 0.556 | down | Isocitrate lyase |
| PHATRDRAFT_26382 | B7FVE3 | 236 | 0.521 | down | Predicted protein |
| PHATR_13358 | B5Y3C9 | 74 | 0.495 | down | Predicted protein |
| PHATRDRAFT_44488 | B7FUA4 | 278 | 0.476 | down | Predicted protein |
| PHATRDRAFT_42543 | B7FRN6 | 153 | 0.463 | down | Predicted protein in |
| PHATRDRAFT_11014 | B7FTR6 | 648 | 0.461 | down | Acyl-coenzyme A dehydrogenase |
| PHATR_44208 | B5Y5Q0 | 511 | 0.457 | down | Predicted protein |
| FABFa | B7G2W2 | 226 | 0.455 | down | 3-oxoacyl-[acyl-carrier-protein] synthase |
| PHATR_43954 | B5Y4W2 | 584 | 0.435 | down | Predicted protein |
| PHATRDRAFT_47663 | B7G4G7 | 457 | 0.435 | down | Predicted protein |
| PHATRDRAFT_11735 | B7FX87 | 99 | 0.418 | down | Predicted protein |
| PHATRDRAFT_42675 | B7FP52 | 546 | 0.413 | down | Predicted protein |
| PHATRDRAFT_48300 | B7G6N6 | 176 | 0.41 | down | Predicted protein |
| PHATRDRAFT_32401 | B7FR26 | 434 | 0.396 | down | Predicted protein |
| PHATRDRAFT_49563 | B7GB18 | 913 | 0.395 | down | Beta-N-acetylhexosaminidase |
| PHATRDRAFT_49647 | B7GBB7 | 207 | 0.392 | down | Predicted protein |
| PHATRDRAFT_54534 | B7G0C3 | 226 | 0.384 | down | Tubulin alpha chain |
| ftsH | A0T0F2 | 615 | 0.36 | down | ATP-dependent zinc metalloprotease FtsH |
| PHATRDRAFT_33928 | B7FU92 | 121 | 0.35 | down | Predicted protein |
| PHATRDRAFT_49837 | B7GC07 | 387 | 0.349 | down | Predicted protein |
| PHATRDRAFT_51291 | B7GED8 | 77 | 0.349 | down | Predicted protein |
| PHATRDRAFT_46983 | B7G219 | 179 | 0.344 | down | Predicted protein |
| PHATRDRAFT_24474 | B7FP06 | 167 | 0.341 | down | Proteasome subunit alpha type |
| PHATRDRAFT_42426 | B7FR90 | 333 | 0.33 | down | Predicted protein |
| PHATRDRAFT_48694 | B7G7E3 | 305 | 0.311 | down | Homoserine dehydrogenase |
| PHATRDRAFT_46529 | B7G1J1 | 149 | 0.304 | down | Predicted protein |
| PHATRDRAFT_12379 | B7FYY0 | 129 | 0.298 | down | Predicted protein |
| PHATRDRAFT_34028 | B7FUI8 | 37 | 0.297 | down | Predicted protein |
| PHATR_21122 | B5Y3W7 | 14 | 0.293 | down | Tubulin beta chain |
| Lhcr14 | B7G503 | 173 | 0.293 | down | Protein fucoxanthin chlorophyll a/c protein |
| PHATRDRAFT_40880 | B7GCN5 | 284 | 0.276 | down | Agmatinase |
| PetJ | B5Y578 | 103 | 0.263 | down | Cytochrome c-553 |
| PHATRDRAFT_44586 | B7FUK9 | 279 | 0.262 | down | Predicted protein |
| PHATRDRAFT_47413 | B7G3A6 | 375 | 0.261 | down | Predicted protein |
| PHATRDRAFT_47589 | B7G468 | 325 | 0.258 | down | Predicted protein |
| psbC | A0T096 | 380 | 0.255 | down | Photosystem II CP43 reaction center protein |
| PHATRDRAFT_47973 | B7G5J6 | 37 | 0.249 | down | Predicted protein |
| PHATRDRAFT_9400 | B7FPT3 | 40 | 0.246 | down | Peptidylprolyl isomerase |
| PHATRDRAFT_8044 | B7FXS1 | 97 | 0.246 | down | Peptidylprolyl isomerase |
|  | A0T096 | 325 | 0.238 | down | Photosystem II CP43 reaction center protein |
| ACS1 | B7FYK0 | 122 | 0.235 | down | Long chain acyl-coa synthetase |
| GapC2a | B7G6K6 | 205 | 0.235 | down | Glyceraldehyde-3-phosphate dehydrogenase |
| PHATRDRAFT_45287 | B7FX53 | 38 | 0.221 | down | Predicted protein |
| PHATRDRAFT_23552 | B7GC36 | 378 | 0.218 | down | Proton-translocating NAD(P)(+) transhydrogenase |
|  | B7FPT3 | 72 | 0.211 | down | Peptidylprolyl isomerase |
| PHATRDRAFT_48723 | B7G855 | 444 | 0.206 | down | Predicted protein |
| PsbO | B7FZ96 | 68 | 0.201 | down | Oxygen-evolving enhancer protein 1 |
| Atp1 | B7G532 | 189 | 0.199 | down | ATP synthase subunit alpha |
| CHC | B7G4Y3 | 146 | 0.186 | down | Clathrin heavy chain |
| PHATR_46937 | B5Y495 | 25 | 0.183 | down | Predicted protein |
| PHATRDRAFT_27923 | B7G162 | 320 | 0.178 | down | H(+)-transporting two-sector ATPase |
| PHATRDRAFT_48519 | B7G7J3 | 519 | 0.17 | down | Predicted protein |
| PHATRDRAFT_30315 | B7GAG0 | 136 | 0.17 | down | Predicted protein |
| CHC | B7G4Y3 | 151 | 0.169 | down | Clathrin heavy chain |
| PHATRDRAFT_40880 | B7GCN5 | 263 | 0.165 | down | Agmatinase |
| PHATRDRAFT_45591 | B7FY71 | 154 | 0.155 | down | Predicted protein |
| PHATR_46710 | B5Y3J4 | 557 | 0.14 | down | Predicted protein |
| PtAP1/2beta | B7S4C6 | 324 | 0.138 | down | Predicted protein |
| CPN60_1 | B7FQ72 | 149 | 0.136 | down | Mitochondria-targeted chaperonin |
| GLNA | B7G6Q6 | 352 | 0.131 | down | GLNA, glutamine synthase |
| PHATRDRAFT_44902 | B7FW24 | 76 | 0.127 | down | Predicted protein |
| PHATRDRAFT_17504 | B7FPI1 | 604 | 0.118 | down | Predicted protein |
| PHATRDRAFT_50437 | B7GE44 | 71 | 0.107 | down | Predicted protein |
| rbcL | Q9TK52 | 313 | 0.094 | down | Ribulose bisphosphate carboxylase large chain |
| psbA | A0T0G9 | 266 | 0.082 | down | Photosystem II protein D1 |
| PHATRDRAFT_44603 | B7FUM8 | 54 | 0.082 | down | Predicted protein |
| FABG | B7G1R8 | 172 | 0.063 | down | 3-oxoacyl-[acyl-carrier protein |
| GPI_1 | B7GDK9 | 472 | 0.047 | down | Glucose-6-phosphate isomerase |
| PHATRDRAFT_22774 | B7G8J0 | 108 | 0.042 | down | Predicted protein |
| rbcL | Q9TK52 | 290 | 0.042 | down | Ribulose bisphosphate carboxylase large chain |
| psbA | A0T0G9 | 267 | 0.037 | down | Photosystem II protein D1 |
| PHATRDRAFT_30145 | B7G9P5 | 163 | 0.026 | down | Citrate synthase |
| GLNA | B7G6Q6 | 353 | 0.025 | down | GLNA, glutamine synthase |
| GLNA | B7G6Q6 | 109 | 0.021 | down | GLNA, glutamine synthase |

Table S3. Differentially N-glycosylated proteins in PtFucT1 knockout mutant

| **Gene** | **Protein**  **Accession** | **Position** | **Fold Change** | **State** | **Description** |
| --- | --- | --- | --- | --- | --- |
| Lhcr6 | B7G4U8 | 146 | 11.248 | up | Protein fucoxanthin chlorophyll a/c protein |
| rbcL | Q9TK52 | 442 | 9.64 | up | Ribulose bisphosphate carboxylase large chain |
| PHATR_46937 | B5Y495 | 25 | 6.789 | up | Predicted protein |
| PHATRDRAFT_47068 | B7G2C0 | 112 | 5.235 | up | Predicted protein |
| PHATR_33351 | B5Y520 | 283 | 3.945 | up | Predicted protein |
| Trx-h2 | B7G7L6 | 130 | 3.587 | up | Thioredoxin h |
| PHATR_52108 | B5Y3K7 | 178 | 3.399 | up | Alpha-mannosidase |
| PHATRDRAFT_43187 | B7FQX7 | 84 | 3.081 | up | Predicted protein |
| PHATR_46710 | B5Y3J4 | 614 | 3.008 | up | Predicted protein |
| PHATRDRAFT_45403 | B7FXK5 | 149 | 2.745 | up | Predicted protein |
| PHATR_44208 | B5Y5Q0 | 367 | 2.659 | up | Predicted protein |
| rbcL | Q9TK52 | 37 | 2.494 | up | Ribulose bisphosphate carboxylase large chain |
| PHATRDRAFT_21548 | B7G341 | 435 | 2.38 | up | Predicted protein |
| PHATRDRAFT_50500 | B7GEA6 | 162 | 2.354 | up | Predicted protein |
| PHATRDRAFT_55162 | B7GDF5 | 213 | 2.343 | up | Predicted protein |
| VDE | B7FUR6 | 397 | 2.218 | up | Violaxanthin deepoxidase |
| FbaC2 | B7G9G9 | 384 | 2.126 | up | Fructose-bisphosphate aldolase |
| PHATR_33450 | B5Y5B6 | 338 | 1.94 | up | Predicted protein |
| PHATRDRAFT_49426 | B7GAJ6 | 118 | 1.913 | up | Predicted protein |
| PHATRDRAFT_42990 | B7FQ92 | 189 | 1.736 | up | Predicted protein |
| PHATRDRAFT_34522 | B7FV27 | 38 | 1.709 | up | Predicted protein |
| PHATRDRAFT_44488 | B7FUA4 | 278 | 0.617 | down | Predicted protein |
| CHC | B7G4Y3 | 665 | 0.595 | down | Isocitrate lyase |
| PHATRDRAFT_51088 | B7G518 | 342 | 0.508 | down | Isocitrate lyase |
| PHATRDRAFT_46983 | B7G219 | 179 | 0.479 | down | Predicted protein |
| PHATRDRAFT_43694 | B7FT54 | 403 | 0.47 | down | Predicted protein |
| PHATRDRAFT_49647 | B7GBB7 | 207 | 0.388 | down | Predicted protein |
| PHATRDRAFT_34028 | B7FUI8 | 37 | 0.36 | down | Predicted protein |
| PHATRDRAFT_43374 | B7FS16 | 509 | 0.358 | down | 9-cis-epoxycarotenoid dioxygenase |
| PHATRDRAFT_35461 | B7FYK4 | 253 | 0.326 | down | Predicted protein |
| PHATRDRAFT_47612 | B7G4A0 | 663 | 0.325 | down | Predicted protein |
| ACC1 | B7G7S4 | 1148 | 0.309 | down | Acetyl-CoA carboxylase |
| PHATRDRAFT_40880 | B7GCN5 | 284 | 0.294 | down | Agmatinase |
| PHATR_44091 | B5Y5B4 | 141 | 0.252 | down | Predicted protein |
| PHATRDRAFT_51092 | B7G5A1 | 385 | 0.25 | down | Glutamine synthetase |
| PHATRDRAFT_23629 | B7GCD8 | 496 | 0.223 | down | Predicted protein |
| PHATRDRAFT_49425 | B7GAJ5 | 151 | 0.213 | down | Predicted protein |
| PHATRDRAFT_23748 | B7GCW6 | 22 | 0.159 | down | Predicted protein |
| PHATR_44054 | B5Y576 | 134 | 0.151 | down | Predicted protein |
| PHATRDRAFT_27757 | B7G0L4 | 213 | 0.133 | down | Predicted protein |
| PHATRDRAFT_46206 | B7G0F8 | 118 | 0.122 | down | Predicted protein |
