# Supplementary material for: Golgi fucosyltransferase 1 reveals its important role in α-1,4-fucose modification of N-glycan in CRISPR/Cas9 diatom Phaeodactylum tricornutum
Source: Microb Cell Fact. 2023 Jan 7;22:6. doi: 10.1186/s12934-022-02000-2 (PMC9826595; doi:10.1186/s12934-022-02000-2)
Supplement: Supplementary file 1 — Additional file 1: Figure S1. The relative expression of PtFucT1 gene in PtFucT1 mutants. Figure S2. The cell density of wild type and PtFucT1 mutants. Figure S3. Gene Ontology (GO) functional annotation of differentially N-glycosylation modification proteins in PtFucT1 mutants. Figure S4. Clusters of Orthologous Groups of protein (COG) functional annotation of differentially N-glycosylation modification proteins in PtFucT1 mutants. Figure S5. Kyoto Encyclopedia of Genes and Genomes (KEGG) functional annotation of differentially N-glycosylation modification proteins in PtFucT1 mutants. Figure S6. Subcellular localization of differentially N-glycosylation modification proteins in PtFucT1-OE (A) and PtFucT1-KO (B) mutants. Figure S7. N-glycan structures on the 19th amino acid of the predicted protein (B7G280) from wild type (A and B) and PtFucT1-KO mutant (C and D). Figure S8. N-glycan structures of putative Acetyl-CoA carboxylase (B7GEB5) from wild type (A) and PtFucT1-KO mutant (B). [file 12934_2022_2000_MOESM1_ESM.docx]

Golgi fucosyltransferase 1 reveals its role in α-1,4-fucose modification of N-glycan in Diatom *Phaeodactylum tricornutum*

Xihui Xie, Jianchao Yang, Hong Du, Jichen Chen, Edmond Sanganyado, Yangmin Gong, Wanna Wang, Weizhou Chen, Zhengyi Liu, Xiaojuan Liu

Figure S1. The relative expression of PtFucT1 gene in PtFucT1 mutants.

Figure S2. The cell density of wild type and PtFucT1 mutants.

Figure S3. Gene Ontology (GO) functional annotation of differentially N-glycosylation modification proteins in PtFucT1 mutants.

Figure S4. Clusters of Orthologous Groups of protein (COG) functional annotation of differentially N-glycosylation modification proteins in PtFucT1 mutants.

Figure S5. Kyoto Encyclopedia of Genes and Genomes (KEGG) functional annotation of differentially N-glycosylation modification proteins in PtFucT1 mutants.

Figure S6. Subcellular localization of differentially N-glycosylation modification proteins in PtFucT1-OE (A) and PtFucT1-KO (B) mutants.

Figure S7. N-glycan structures on the 19^th^ amino acid of the predicted protein (B7G280) from wild type (A and B) and PtFucT1-KO mutant (C and D).

Figure S8. N-glycan structures of putative Acetyl-CoA carboxylase (B7GEB5) from wild type (A) and PtFucT1-KO mutant (B).

Figure S1. The relative expression of PtFucT1 gene in PtFucT1 mutants.

WT, wild type; OE, PtFucT1 overexpression mutants; KO, PtFucT1 knockout mutants; ND, not detected.

Figure S2. The cell density of wild type and PtFucT1 mutants.

WT, wild type; OE, PtFucT1 overexpression mutants; KO, PtFucT1 knockout mutants.

Figure S3. Gene Ontology (GO) functional annotation of differentially N-glycosylated modification proteins in PtFucT1 mutants. Three main classes: Biological Process (BP), Cellular Component (CC) and Molecular Function (MF). A, Go annotation in PtFucT1-OE mutant; B, Go annotation in PtFucT1-KO mutant.

Figure S4. Clusters of Orthologous Groups of protein (COG) functional annotation of differentially N-glycosylated modification proteins in PtFucT1 mutants. A, COG annotation in PtFucT1-OE mutant; B, COG annotation in PtFucT1-KO mutant.

Figure S5. Kyoto Encyclopedia of Genes and Genomes (KEGG) functional annotation of differentially N-glycosylated modification proteins in PtFucT1 mutants. Four main classes: Genetic Information Processing, Environmental Information Processing, Cellular Processes, Metabolism. A, KEGG annotation in PtFucT1-OE mutant; B, KEGG annotation in PtFucT1-KO mutant.

Figure S6. Subcellular localization of differentially N-glycosylated modification proteins in PtFucT1-OE (A) and PtFucT1-KO (B) mutants. Chlo, chloroplast; cyto, cytosol; nucl, nucleus; plas, plasma membrane; mito, mitochondria; extr, extracellular; vacu, vacuolar membrane.

Figure S7. N-glycan structures on the 19^th^ amino acid of the predicted protein (B7G280) from wild type (A and B) and PtFucT1-KO mutant (C and D). Yellow circle, galactose; yellow star, Xylose; red triangle, fucose; green circle, mannose; blue circle, glucose; blue square, N-acetylglucosamine.

Figure S8. N-glycan structures of putative Acetyl-CoA carboxylase (B7GEB5) from wild type (A) and PtFucT1-KO mutant (B). Yellow star, Xylose; red triangle, fucose; green circle, mannose; blue square, N-acetylglucosamine.
